# Dynamically predicting renal failure after development of diabetes across biobanks

**DOI:** 10.1371/journal.pdig.0001375

**Published:** 2026-05-04

**Authors:** Aubrey Jensen, Sayera Dhaubhadel, Jonathan Hori, Nabil Alami, Gang Li, Hua Zhou, Sridharan Raghavan, Benjamin H. McMahon, Peter Reaven, Jin J. Zhou

**Affiliations:** 1 Department of Biostatistics, University of California, Los Angeles, California, United States of America; 2 Phoenix VA Health Care System, Phoenix, Arizona, United States of America; 3 Los Alamos National Laboratory, Los Alamos, New Mexico, United States of America; 4 CentraleSupélec, Université Paris-Saclay, Gif-sur-Yvette, France; 5 Section of Academic Primary Care, United States of America Department of Veterans Affairs Eastern Colorado Health Care System, Aurora, Colorado, United States of America; 6 Division of General Internal Medicine, University of Colorado Anschutz Medical Campus, Aurora, Colorado, United States of America; Liverpool John Moores University - City Campus: Liverpool John Moores University, UNITED KINGDOM OF GREAT BRITAIN AND NORTHERN IRELAND

## Abstract

End-stage renal disease (ESRD) remains a major complication of diabetes, yet existing static risk scores may lose accuracy as patient profiles evolve and competing mortality risks change over time. We developed and externally validated a landmark-based ESRD Dynamic Risk Score (ESRD-DRS) that updates individual risk estimates at 1, 5, and 10 years after diabetes diagnosis using routinely collected EHR data. We assembled a retrospective cohort of 708,435 U.S. Veterans with newly diagnosed diabetes in the Veterans Health Administration (VHA). At 1, 5, and 10 years after diabetes diagnosis (LM1, LM5, and LM10), we fit penalized Fine–Gray subdistribution hazard models, drawing from more than 400 demographic, medication, comorbidity, and laboratory variables. Models were evaluated over 1-, 5-, and 10-year horizons for discrimination (area under the time-dependent receiver operating characteristic curve [AUROC]) and calibration (Brier score), and compared with the established RECODe and 4-variable KFRE risk equations. External validation was performed in an independent All of Us (AoU) cohort (n = 13,223). In the VHA cohort (median follow-up 7.6 years), 8,955 patients (1.26%) developed ESRD, and 136,666 (19.3%) died without ESRD. At LM1, ESRD-DRS achieved AUROCs of 0.93, 0.90, and 0.85 for 1-, 5-, and 10-year risk, respectively, and Brier scores ranging from 0.00098 to 0.0162. In the AoU cohort, corresponding AUROCs were 0.94, 0.91, and 0.86, with similar calibration performance. RECODe and KFRE yielded lower discrimination and poorer calibration. Top risk predictors, including estimated glomerular filtration rate, albuminuria, systolic blood pressure, and age, were consistent across landmarks and cohorts. ESRD-DRS, a scalable landmark approach that accounts for competing mortality and evolving patient profiles, outperformed existing static equations. Embedding ESRD-DRS into EHR workflows may support more timely, individualized ESRD risk assessment in patients with diabetes.

## Author summary

We asked whether we could better identify people with newly diagnosed diabetes who are most likely to develop kidney failure in the future. Existing risk calculators are usually built from a single clinic visit, often require laboratory tests that are not always done early in diabetes, and rarely account for the fact that many people die of other causes before kidney failure occurs.

We used routinely collected electronic health records from more than 700,000 United States veterans with diabetes and from over 13,000 participants in the National Institutes of Health All of Us program. We built a kidney failure risk score that updates at 1, 5, and 10 years after diabetes diagnosis, using information that is already stored in the health record, such as age, kidney function, urine protein, blood pressure, medicines, and other conditions.

Our dynamic score predicted kidney failure more accurately and stayed better aligned with observed outcomes over time than two established diabetes risk equations. If implemented in health record systems, it could help clinicians and patients decide when to start kidney-protective treatments and when to refer to kidney specialists, with the goal of preventing or delaying kidney failure.

## Introduction

Although diabetes care has advanced significantly over the past few decades [1–4], serious microvascular and macrovascular complications remain common. Among these, end-stage renal disease (ESRD) is particularly devastating: It drives substantial morbidity and mortality, impairs quality of life, and incurs high healthcare costs [5,6]. Early identification of patients with diabetes who are most likely to progress to ESRD is therefore essential for timely, targeted intervention, especially given an expanding array of therapies that can delay or even prevent kidney failure. Yet predicting ESRD risk is challenging. Multiple pathophysiological pathways and comorbid conditions shape renal decline, and the substantial competing risk of death can cause standard survival analyses to overestimate ESRD incidence, particularly in older, multimorbid populations and over long time horizons [7].

Several ESRD risk equations have been proposed [8–12]. The Kidney Failure Risk Equation (KFRE) performs well in general chronic kidney disease (CKD) populations [13], and RECODe (Risk Equations for Complications Of type 2 Diabetes) provides complication-specific risk estimates among people with type 2 diabetes (T2D) [14]. However, model performance often diminishes when these tools are applied in external settings or in broader, more heterogeneous patient cohorts [15]. Two features of existing models likely contribute to this loss of performance. First, many require biomarkers that are infrequently measured early in the course of diabetes, limiting applicability in routine care. Second, most models are static: they are fit at a single baseline time point and do not update to reflect evolving clinical profiles or shifts in background risk over time. Both time-varying risk factor effects and changes in the underlying cumulative incidence can distort long-term risk estimates and degrade calibration. A dynamic, EHR (electronic health records)-embedded risk tool that relies on routinely collected data, explicitly accounts for competing mortality, and is designed to update across the diabetes care trajectory could address these limitations and better support risk-directed renal prevention.

**Fig 1:**
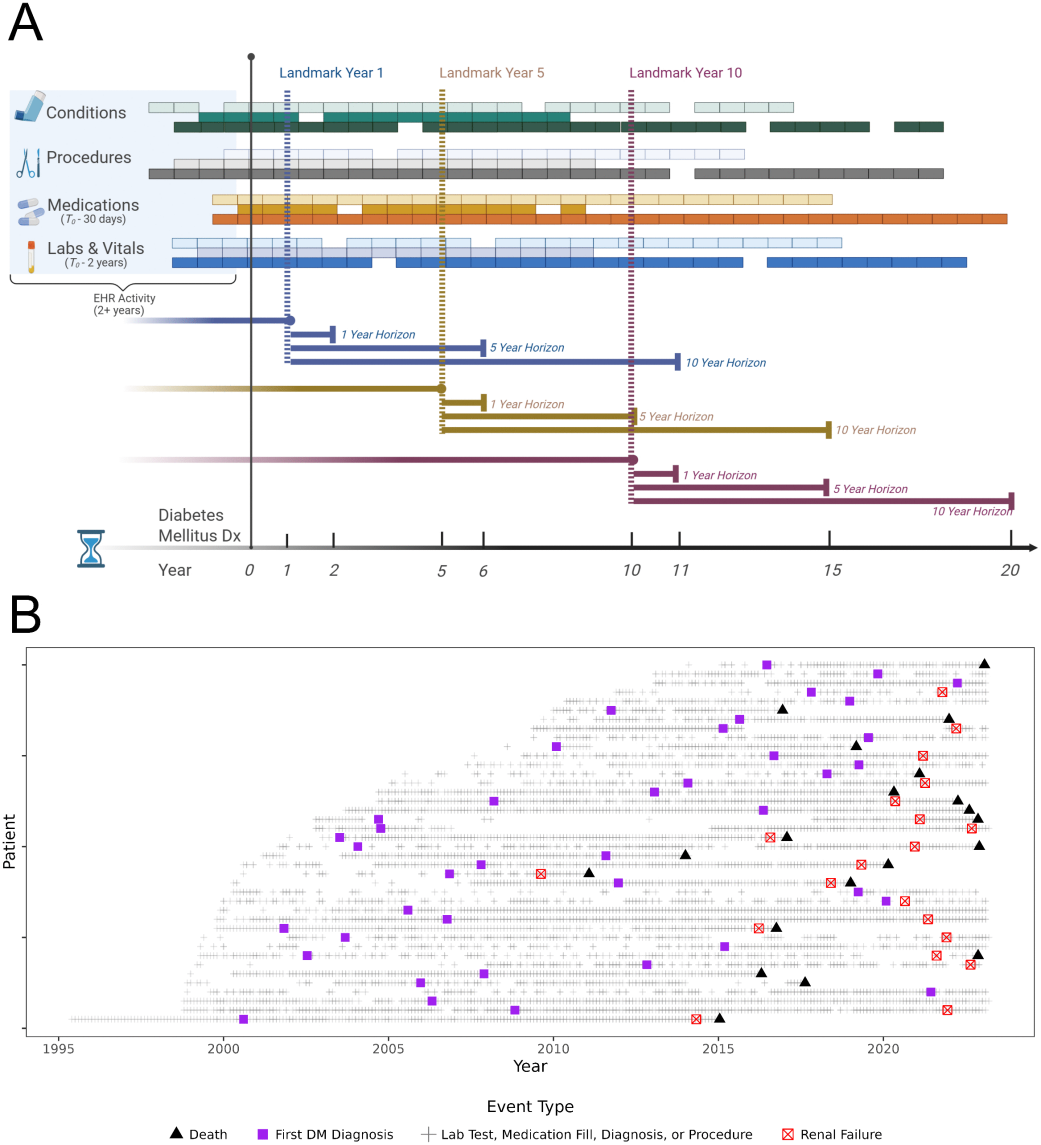
Study design schema. **A**: Patients who have at least 1 visit in each of the two years prior to diabetes qualify to enter the cohort. The date of the first diabetes code or medication (excluding metformin) is the index date. We also require patients to have ≥ 1 low-density lipoprotein (LDL) cholesterol, ≥ 1 hemoglobin A1c, and ≥ 1 estimated glomerular filtration rate (eGFR) after the index date. We define time at risk to be the time after the index date to the date patients exit the cohort. **B**: 20 examples of patients’ EHR trajectories in VHA, including diabetes diagnosis (Dx), renal failure, and death.

Our aim was to address these gaps by developing the ESRD Dynamic Risk Score (ESRD-DRS), a model that accounts for competing risk and updates risk estimates at 1, 5, and 10 years after diabetes diagnosis (**Fig 1A**). Our statistical framework combines Fine-Gray sub-distribution (FG SDH) hazard regression with a scalable linear search algorithm and a minimax concave penalty (MCP) to select parsimonious predictors from more than 400 EHR-derived variables while properly accounting for competing mortality risk. In contrast to prior ESRD tools that are static, rely on a small set of baseline predictors, or have limited external validation, ESRD-DRS is built from a very large, real-world national cohort of newly diagnosed diabetes patients and is explicitly designed for repeated use over the course of care. We benchmark its performance against established diabetes risk equations (RECODe and KFRE), evaluate transportability in an independent biobank (All of Us [AoU]), and dissect the relative contribution of refitting versus simple recalibration of the baseline hazard to maintain long-term calibration. Together, these features make ESRD-DRS a novel, scalable template for dynamic kidney-failure risk prediction and a practical candidate for integration into EHR workflows to support proactive, individualized renal care.

## Results

### VHA diabetes cohort

There were 708,435 patients newly diagnosed with diabetes within the Veterans Health Administration (VHA) who met study criteria at Landmark 1 (LM1, defined in **MATERIALS AND METHODS** section below, **S1 Fig**), of which 7% were female. The majority (67.9%) of the cohort was White, 22% were Black, and 6.3% were Hispanic of any race. The median (interquartile range [IQR]) age at the first diagnosis of diabetes for patients in the VHA cohort was 59.9 (53.1 - 65.8) years. Follow-up times ranged from 0.1 to 24.3 years, with a median (IQR) of 7.6 (3.9-12.1) years (**Table A in** **S1 Tables**). There were 8,955 (1.26%) incident ESRD cases and 136,666 (19.3%) deaths without ESRD during follow-up. Examples of patient timelines are illustrated in **Fig 1B**. The median (IQR) time to ESRD and the time to death without ESRD were 7.5 (3.6 – 12.0) years and 7 (3.6 - 11.1) years, respectively. The estimated cumulative incidence function (CIF) of ESRD increased over increasing horizon years after each landmark time (**Fig A in** **S2 Fig** **and Table B in** **S1 Tables**). Patients with lower estimated glomerular filtration rate (eGFR) (<60 mL/min/1.73m²) at LM1 and Black/African Americans had a significantly higher ESRD incidence rate over time (**Fig A in** **S2 Fig**). In comparison to ESRD, the CIF for death was much higher, with male and White patients exhibiting the highest death rate (**Fig B in** **S2 Fig**). At LM1, median (IQR) Body Mass Index (BMI) was 32.3 (28.7 - 36.6) kg/m^2^, hemoglobin A1c (HbA1c) 6.3% (5.9% - 6.8%) (**Table A in** **S1 Tables** and **S3 Fig**). 7.7% were receiving insulin treatment, 35.4% were on metformin, 42.6% were on ACE (angiotensin-converting enzyme) Inhibitors, and 34.8% were on beta blockers (**S1** **Methods** and **Table A in** **S1 Tables**).

### Dynamic prediction models and their performances in the VHA diabetes cohort

Out of the 430 features generated, we aggregated and included 227 from several domains in the VHA cohort for training and internal validation. These included 58 conditions (**Table C in** **S1 Table**), 14 procedures (**Table C in** **S1 Table**), 49 medications (**Table D in** **S1 Table**), 87 vitals and lab results (43 category levels; **Table E in** **S1 Table** and 44 continuous markers; **S4 Fig** and **Table F in** **S1 Table**), and 19 other features (demographics and other health factors such as smoking, etc.). After the initial screening, 158 features stayed in the model and were further subject to MCP selection. A total of 53 predictors were then selected by MCP at LM1 and included in all three landmark models.

**Table 1** summarizes the ESRD-DRS’s discrimination performance. At LM1, the model's internal validation Area under the time-dependent receiver operating characteristic (ROC) curve (AUROC) (95% CI) were 0.928 (0.90–0.95), 0.896 (0.883–0.909), and 0.850 (0.838–0.86) for 1-, 5-, and 10-year horizons, respectively. By comparison, RECODe’s AUROCs were 0.897 (0.865–0.923), 0.848 (0.830–0.864), and 0.793 (0.778–0.806), and KFRE’s were 0.918 (0.886-0.944), 0.868 (0.852-0.883), and 0.811 (0.797-0.823), respectively. Although the predicted cumulative incidences of ESRD at LM1 were relatively low (0.00103, 0.00511, and 0.012 for 1-, 5-, and 10-year horizons), the area under the precision (i.e., positive predictive value)-recall (i.e., sensitivity) curve (AUPRC) values remained high (0.145, 0.241, and 0.235, respectively), surpassing the corresponding RECODe AUPRC values of 0.131, 0.15, and 0.135, and the KFRE values of 0.111, 0.167, and 0.138, especially for longer horizons. Discrimination performances in other LM years were similar to LM1. Although longer prediction horizons typically yielded lower discrimination performance, the dynamic risk score still demonstrated satisfactory discrimination. For the 1-year risk of ESRD (i.e., Horizon Year 1), our model's AUROC ranged from 0.93 to 0.95; for 5-year ESRD risk (i.e., Horizon Year 5), it was around 0.90; and for the 10-year ESRD risk (i.e., Horizon Year 10), our model’s AUROC was at 0.85.

**Table 1:** AUROC and AUPRC of the Dynamic prediction (ESRD-DRS) model in VHA (Top) and AoU (external validation, Bottom), and benchmark with the RECODe risk model and KFRE. AoU: All of Us; DM: Diabetes Mellitus; AUROC: Area Under the time-dependent Receiver Operating Characteristic Curve (ROC); AUPRC: Area under the Precision-Recall Curve (PRC); LM: Landmark; CIF: cumulative incidence function; ESRD-DRS: End-stage renal disease Dynamic Risk Score; RECODe: Risk Equations for Complications Of type 2 Diabetes; KFRE: Kidney Failure Risk Equation; VHA: Veterans Health Administration.

VHA										
Model	LM Yrs	Horizon Year 1 (1-Year Risk)	Horizon Year 5 (5-Year Risk)	Horizon Year 10 (10-Year Risk)	Horizon Year 1 (1-Year Risk)		Horizon Year 5 (5-Year Risk)		Horizon Year 10 (10-Year Risk)	
		AUROC (95%CI)	AUROC (95%CI)	AUROC (95%CI)	AUPRC^*^	CIF	AUPRC	CIF	AUPRC	CIF
**ESRD-DRS**	**1**	0.928 (0.900-0.950)	0.896 (0.883-0.909)	0.850 (0.838-0.860)	0.145	0.00103	0.241	0.00511	0.235	0.0120
**RECODe**		0.897 (0.865-0.923)	0.848 (0.83-0.8640)	0.793 (0.778-0.806)	0.131	0.00103	0.152	0.00511	0.135	0.0120
**KFRE**		0.918 (0.886-0.944)	0.868 (0.852-0.883)	0.811 (0.797-0.823)	0.111	0.00103	0.167	0.00511	0.138	0.0120
**ESRD-DRS**	**5**	0.952 (0.933-0.970)	0.909 (0.896-0.922)	0.855 (0.844-0.866)	0.140	0.00120	0.274	0.00686	0.237	0.0168
**RECODe**		0.921 (0.891-0.945)	0.857 (0.842-0.870)	0.798 (0.785-0.812)	0.114	0.00120	0.164	0.00686	0.149	0.0168
**KFRE**		0.928 (0.902-0.953)	0.875 (0.859-0.890)	0.792 (0.776-0.807)	0.110	0.00120	0.159	0.00686	0.124	0.0168
**ESRD-DRS**	**10**	0.949 (0.924-0.970)	0.911 (0.898-0.923)	0.856 (0.84-0.8720)	0.213	0.00192	0.300	0.0113	0.256	0.0263
**RECODe**		0.908 (0.867-0.939)	0.866 (0.849-0.882)	0.831 (0.811-0.848)	0.129	0.00192	0.190	0.0113	0.185	0.0263
**KFRE**		0.929 (0.898-0.958)	0.873 (0.855-0.889)	0.790 (0.768-0.812)	0.121	0.00192	0.162	0.0113	0.140	0.0263
**AoU**										
**Model**	**LM Yrs**	**Horizon Year 1** **(1-Year Risk)**	**Horizon Year 5** **(5-Year Risk)**	**Horizon Year 10** **(10-Year Risk)**	**Horizon Year 1** **(1-Year Risk)**		**Horizon Year 5** **(5-Year Risk)**		**Horizon Year 10** **(10-Year Risk)**	
		**AUROC (95%CI)**	**AUROC (95%CI)**	**AUROC (95%CI)**	**AUPRC**	**CIF**	**AUPRC**	**CIF**	**AUPRC**	**CIF**
**ESRD-DRS**	**1**	0.938 (0.902-0.969)	0.912 (0.873-0.949)	0.860 (0.818-0.897)	0.0340	0.00180	0.364	0.00771	0.344	0.0163
**RECODe**		0.869 (0.761-0.948)	0.846 (0.788-0.898)	0.777 (0.726-0.829)	0.0192	0.00180	0.165	0.00771	0.152	0.0163
**KFRE**		0.901 (0.772-0.986)	0.877 (0.822-0.926)	0.813 (0.768-0.863)	0.0436	0.00180	0.293	0.00771	0.270	0.0163
**ESRD-DRS**	**5**	0.849 (0.598-0.999)	0.850 (0.775-0.918)	0.822 (0.759-0.879)	0.250	0.00089	0.316	0.00794	0.337	0.0187
**RECODe**		0.788 (0.490-0.998)	0.774 (0.693-0.846)	0.776 (0.718-0.831)	0.118	0.00089	0.154	0.00794	0.128	0.0187
**KFRE**		0.785 (0.481-0.999)	0.816 (0.728-0.892)	0.779 (0.713-0.848)	0.134	0.00089	0.231	0.00794	0.263	0.0187
**ESRD-DRS**	**10**	0.984 (0.956-0.999)	0.916 (0.855-0.969)	0.901 (0.846-0.947)	0.191	0.00166	0.348	0.0110	0.490	0.0289
**RECODe**		0.950 (0.832-1.00)	0.877 (0.789-0.952)	0.797 (0.714-0.874)	0.304	0.00166	0.262	0.0110	0.333	0.0289
**KFRE**		0.989 (0.968-1.00)	0.856 (0.768-0.939)	0.831 (0.764-0.896)	0.524	0.00166	0.364	0.0110	0.391	0.0289

* Bootstrap confidence interval calculations were excluded owing to their substantial computational demands.

Overall, the ESRD risk score exhibited good calibration across different landmark times and horizon years (**Table 2 and** **S5 Fig**). For instance, at LM1, the Brier scores were 9.8 × 10 ⁻ ⁴, 5.2 × 10 ⁻ ³, and 1.6 × 10 ⁻ ² for the 1-, 5-, and 10-year horizons, respectively. Brier scores slightly increased at later landmark years. In general, calibration slopes were estimated to be greater than 1, indicating that prediction models may underestimate the risk of ESRD. However, this trend improved at later landmark years or horizon years, presumably due to higher observed event rates. For example, at LM1, when predicting 1-year risk, the calibration slope was 1.44 (0.01); at LM5, when predicting 5-year risk, it was 1.18 (0.0076); and at LM10, when predicting 10-year risk, it was 1.02 (0.014). In contrast, RECODe’s calibration slopes were much worse. Out of 9 combinations of 3 LM years and 3 Horizons evaluated (Horizons were defined in the **MATERIALS AND METHODS** section below), only 1 (LM1 and Horizon 1 [H1]) RECODe showed better calibration slopes (1.27 RECODe vs 1.44 ESRD-DRS). The same was true for 2 combinations for KFRE (LM1 and Horizon 10 [H10], 1.05 vs. 1.13; LM10 and Horizon 5 [H5], 0.95 vs. 1.15). Calibration plots (**S5 Fig** **and** **S6 Fig**) further illustrate the good calibration performance.

**Table 2:** Calibration performance, measured by Brier score and calibration plot slopes, of the ESRD Dynamic prediction (ESRD-DRS) model in AoU (external validation) and benchmarked against the RECODe risk model. AoU: All of Us; DM: Diabetes Mellitus; ESRD-DRS: end-stage renal disease dynamic risk score; RECODe: Risk Equations for Complications Of Type 2 Diabetes; KFRE: Kidney Failure Risk Equation; ESRD: end-stage renal disease; VHA: Veterans Health Administration.

VHA								
			Horizon Year 1		Horizon Year 5		Horizon Year 10	
Model	LM Year	Recalibration Method	Slope (SE)	Brier Score	Slope (SE)	Brier Score	Slope (SE)	Brier Score
**ESRD-DRS**	**1**	**Fine-Gray**	1.44 (0.01)	0.000976	1.27 (0.0072)	0.00521	1.13 (0.0072)	0.0162
**RECODe**		**Cox**	1.27 (0.028)	0.00105	1.87 (0.023)	0.00629	1.50 (0.02)	0.0225
		**Fine-Gray**	1.86 (0.045)	0.00105	2.11 (0.031)	0.00583	1.80 (0.025)	0.0177
**KFRE**		**Cox**	2.79 (0.023)	0.00102	1.61 (0.011)	0.00595	1.05 (0.0092)	0.0217
		**Fine-Gray**	0.302 (0.0023)	0.00141	0.317 (0.0021)	0.00793	0.289 (0.0025)	0.0236
**ESRD-DRS**	**5**	**Fine-Gray**	1.13 (0.01)	0.00115	1.18 (0.0076)	0.00758	1.02 (0.0085)	0.029
**RECODe**		**Cox**	0.418 (0.017)	0.00127	1.27 (0.021)	0.00973	1.38 (0.022)	0.0448
		**Fine-Gray**	0.678 (0.028)	0.00124	1.34 (0.026)	0.00857	1.52 (0.025)	0.031
**KFRE**		**Cox**	2.3 (0.02)	0.0012	1.19 (0.0099)	0.00913	0.804 (0.0092)	0.0439
		**Fine-Gray**	0.248 (0.0022)	0.00202	0.28 (0.0023)	0.0128	0.262 (0.003)	0.0419
**ESRD-DRS**	**10**	**Fine-Gray**	1.34 (0.012)	0.00176	1.15 (0.01)	0.0143	1.02 (0.014)	0.0802
**RECODe**		**Cox**	0.545 (0.018)	0.00206	1.24 (0.022)	0.0194	1.29 (0.03)	0.153
		**Fine-Gray**	0.776 (0.025)	0.00199	1.46 (0.028)	0.0160	1.53 (0.035)	0.0833
**KFRE**		**Cox**	1.76 (0.02)	0.00193	0.947 (0.011)	0.0186	0.66 (0.014)	0.153
		**Fine-Gray**	0.245 (0.0029)	0.00335	0.279 (0.0033)	0.0235	0.27 (0.0053)	0.101
**AoU**								
**ESRD-DRS**	**1**	**Fine-Gray**	0.415 (0.034)	0.00189	1.28 (0.023)	0.00762	1.11 (0.026)	0.0239
**RECODe**		**Cox**	0.13 (0.04)	0.00194	1.04 (0.041)	0.00892	1.15 (0.048)	0.0269
		**Fine-Gray**	1.61 (0.2)	0.00186	2.32 (0.11)	0.00903	1.89 (0.091)	0.0265
**KFRE**		**Cox**	1.05 (0.065)	0.00184	1.43 (0.033)	0.00831	1.01 (0.03)	0.0258
		**Fine-Gray**	0.116 (0.0065)	0.00436	0.307 (0.007)	0.0141	0.3 (0.009)	0.0367
**ESRD-DRS**	**5**	**Fine-Gray**	1.14 (0.026)	0.000765	1.04 (0.027)	0.0095	0.920 (0.031)	0.0367
**RECODe**		**Cox**	0.393 (0.029)	0.00095	0.911 (0.037)	0.0105	0.886 (0.048)	0.0406
		**Fine-Gray**	4.49 (0.31)	0.000915	2.17 (0.12)	0.0107	1.68 (0.11)	0.0392
**KFRE**		**Cox**	2.55 (0.1)	0.00089	1.19 (0.042)	0.0103	0.861 (0.038)	0.0399
		**Fine-Gray**	0.176 (0.0068)	0.0022	0.203 (0.0084)	0.0204	0.238 (0.011)	0.0575
**ESRD-DRS**	**10**	**Fine-Gray**	1.35 (0.044)	0.0015	0.954 (0.032)	0.0143	1.18 (0.043)	0.069
**RECODe**		**Cox**	0.673 (0.029)	0.00164	1.00 (0.047)	0.0159	1.34 (0.063)	0.0783
		**Fine-Gray**	1.02 (0.17)	0.00175	1.15 (0.11)	0.0164	1.97 (0.14)	0.0764
**KFRE**		**Cox**	2.66 (0.068)	0.00152	0.915 (0.036)	0.0153	0.803 (0.039)	0.0785
		**Fine-Gray**	0.251 (0.008)	0.004	0.296 (0.012)	0.0248	0.341 (0.018)	0.0927

* In VHA, individual risks were calculated by incorporating a recalculated baseline hazard at each evaluation horizon.

$ Only models with updated weights were exported to AoU for external validation.

& We used the baseline hazard estimated from the VHA cohort for different horizons, then recalibrated using the baseline hazard estimated in AoU.

Model performance and calibration across subgroups are shown in **Table G** (AUROC)/**Table H** (Area under the Precision-Recall Curve (PRC) [AUPRC]) **in** **S1 Tables**, and **Table I in** **S1 Tables** (Brier score and calibration slope)/**S7 Fig** (VHA Subpopulation Calibration Plots), respectively. The general pattern across subgroups is consistent with the whole group, except the confidence intervals of AUCs are much wider (e.g., in females) due to smaller sample sizes (**S8 Fig**). Our model outperforms RECODe and KFRE across most subgroups and metrics. Our ESRD-DRS performed notably better among those with eGFR < 60 mL/min/1.73 m², i.e., among patients with diabetes who have a higher baseline risk of kidney disease.

### Variable importance and model coefficient comparisons

**Fig 2:**
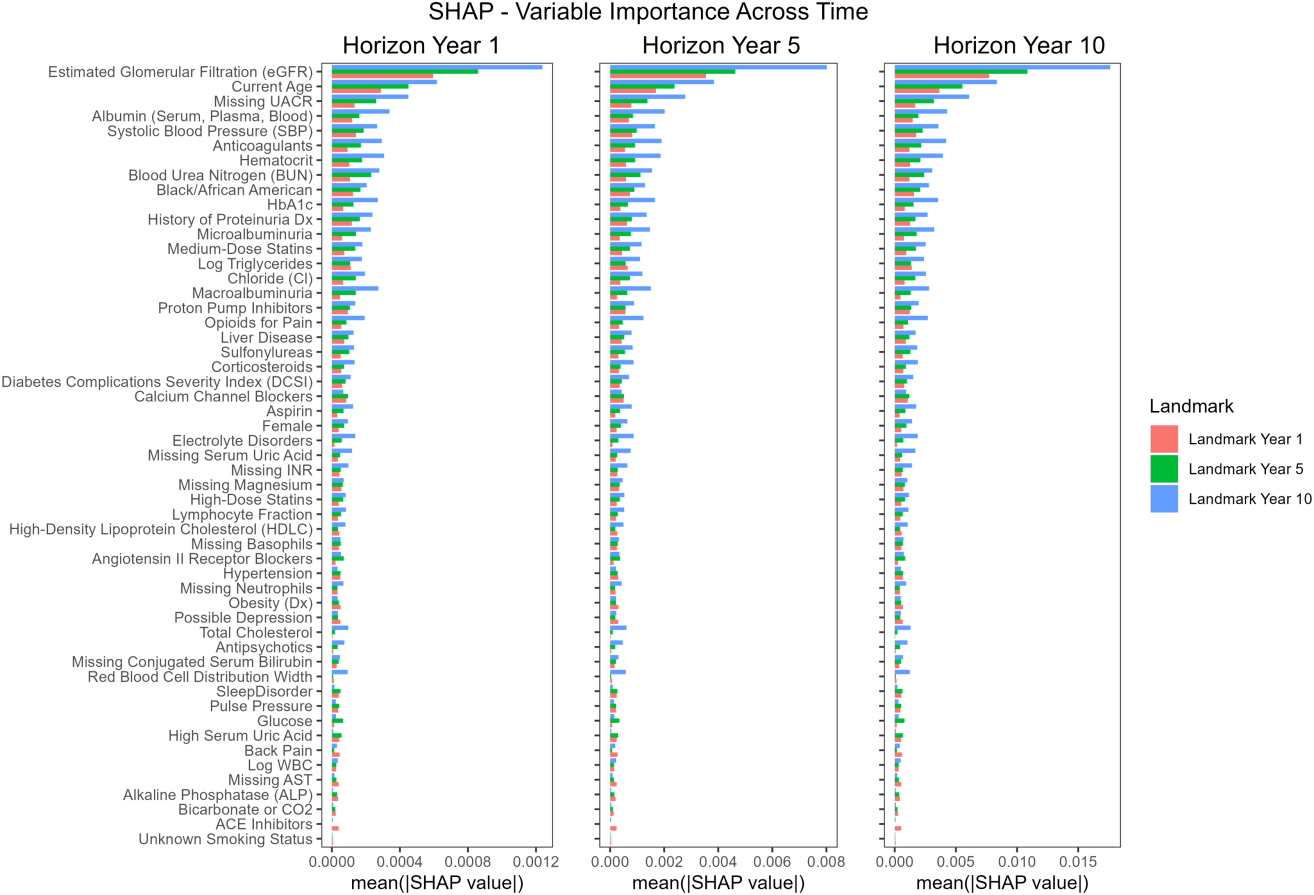
Feature importance as defined by mean absolute SHAP (SHapley Additive exPlanations) value. Importance estimates vary across time and are shown for three landmark years and three horizon times.

**Fig 2** and **Table J in** **S1 Tables** show variable importance determined by SHAP values. The most influential predictors included eGFR, age, systolic blood pressure (SBP), categorized Urine Albumin-to-Creatinine Ratio (UACR), and Black race. The importance of eGFR as a predictor increased at later landmarks, as did many other important predictors, such as categorized UACR. Individual SHapley Additive exPlanations (SHAP) value plots for the top 10 important features among ESRD patients are illustrated in **S9 Fig**, while those for all patients are shown in **S10 Fig**. Although the feature importances varied slightly across different LMs and horizons, the top 10 features remained largely consistent. Notably, anticoagulant usage emerged as a significant feature in LM5 and LM10, associated with a decreased risk of ESRD in the overall patient population (**S10 Fig**).

### External validation in the AoU diabetes cohort

#### AoU cohort characteristics.

There were 13,223 patients with newly diagnosed diabetes in the AoU cohort at LM1, of whom 64% were female, 51.5% were White, 24.6% were Black, and 15.5% were Hispanic. The median (IQR) age at first diabetes diagnosis of patients was 56.2 (47.8 – 64.1) years, slightly younger than those in VHA. The follow-up time ranged from 0.1 to 39.5 years, with a median (IQR) of 8.1 (4.5-12.9) years. There were 219 (1.66%) ESRD cases and 372 (2.81%) deaths without ESRD during follow-up. The median times to (IQR) ESRD and death without ESRD event were 7.2 (2.8 – 12.8) and 8.5 (4.3 – 13.4) years, respectively. All the necessary features in VHA were defined on the AoU platform (**Tables C, D, and E in** **S1 Tables**). Cohort characteristics for LM1 in the AoU cohort are shown in **Table A in** **S1 Tables**. At LM1, 9.39% were on insulin, 20.3% on metformin, 13.9% on ACE inhibitors, and 15.2% on beta blockers. The number of patients and events at each landmark and horizon time was detailed in **Table K in** **S1 Tables**. Cardiometabolic metrics fall within a healthier range compared to those in VHA (**Table A in** **S1 Tables** **and** **S3 Fig**).

#### Model performance in AoU.

The discrimination performance of ESRD-DRS in AoU remained high and superior to that of RECODe and KFRE (**Table 1**, bottom panel). At LM1, AUROCs (95% CI) for ESRD-DRS were 0.938 (0.902-0.969), 0.912 (0.873-0.949), and 0.860 (0.818-0.897) for 1-, 5-, and 10-year horizons, respectively. In comparison, AUROCs for the RECODe score in AoU were 0.869 (0.761-0.948), 0.846 (0.788-0.898), and 0.777 (0.726-0.829), and AUROCs for the KFRE score were 0.901 (0.772-0.986), 0.877 (0.822-0.926), and 0.813 (0.768-0.863). As for calibration (**Table 2 and Table I in** **S1 Table**), 5-year risk (H5) and 10-year risk (H10) showed a statistically acceptable calibration for all landmark years, while 1-year risk (H1) was less consistent, largely due to the very small number of observed events during this short horizon period.

**Fig 3:**
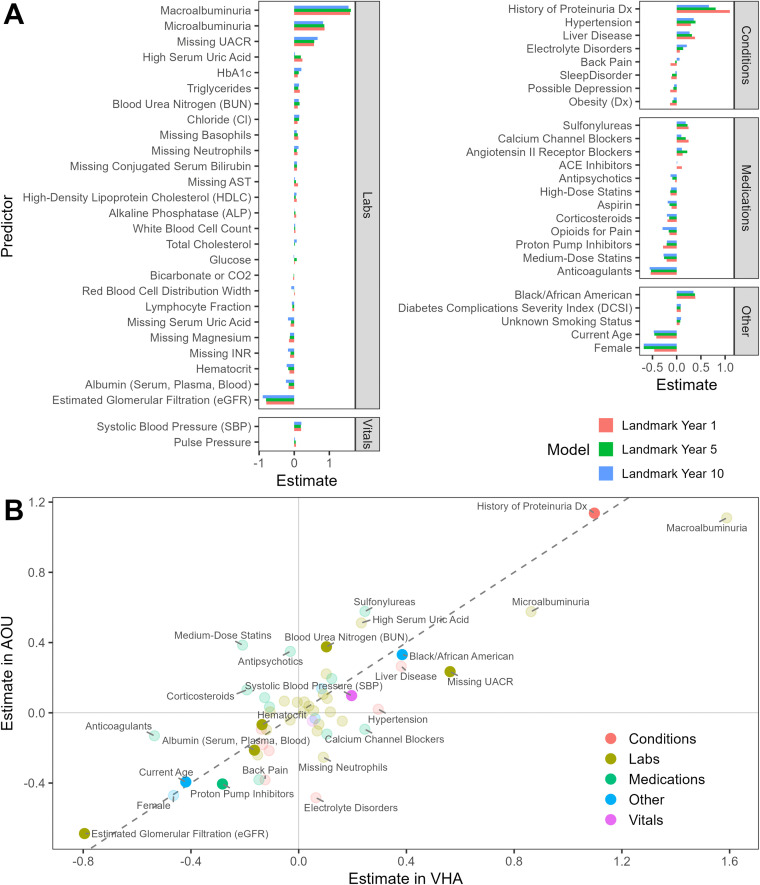
Comparisons of model coefficients across. (A) three landmark models fit in VHA (Veterans Health Administration) diabetes patients. (B) VHA vs. AoU (All of Us) at landmark year 1. **The Fine-Gray sub-distribution model coefficients were re-estimated in AoU using the predictors selected in VHA. Features with effect size >0.4, along with the top 10 most important features by mean absolute SHAP value at any horizon time, are shown in solid points.**

The model coefficients estimated from the VHA data across landmark times remained similar and closely aligned with the SHAP variable importance values (**Fig 3A and Table L in** **S1 Table**). Interestingly, the feature coefficients estimated from the VHA and AoU biobanks showed high consistency between the two cohorts (**Fig 3B and Table L in** **S1 Table**).

### Optimal thresholds for clinical decision-making

Optimal thresholds for predicted probability of ESRD identified by Youden’s J and Top-Left methods are presented in **Table M in** **S1 Table**. For example, at LM1 and for H10, using Youden’s J, the optimal threshold was 0.153%, yielding a sensitivity of 0.702 and specificity of 0.856. Using the Top-Left method, the optimal threshold was 0.198%, corresponding to a sensitivity of 0.766 and specificity of 0.777. Similar trade-offs between sensitivity and specificity were observed between the two methods at other landmarks and horizon years.

## Discussion

In this study, we developed and externally validated a landmark-based, dynamic prediction model for ESRD in patients with newly diagnosed diabetes. Rather than relying on a single, static baseline score, our framework refits FG SDH models at 1, 5, and 10 years after diagnosis, updating risk estimates to reflect evolving predictors and the competing risk of death. Leveraging high-dimensional EHR data with variable selection, the model recalibrates at natural clinical milestones. It achieved excellent discrimination and calibration, outperformed static tools such as RECODe and KFRE, and has the potential to integrate seamlessly into routine visits to support timely, personalized interventions (for example, initiating sodium–glucose cotransporter 2 (SGLT2) inhibitors or intensifying blood pressure control). The existing ESRD prediction tools achieve high discrimination in development cohorts but often show worse calibration when applied externally (**S5 Fig** **and** **S6 Fig**). By contrast, our landmark framework preserves both discrimination and calibration across diverse populations and over decades of follow-up. Dynamic prediction via landmarking refits FG SDH models at pre-specified times using updated covariates, capturing evolving patient profiles and competing mortality without complex joint modeling [16–18]. This approach improves calibration and reduces bias versus single-origin models [16] and was critical in our cohort, where ~20% died before ESRD, to avoid the ESRD risk overestimation seen with standard Cox models [19].

To disentangle the relative gains from refitting the entire model (including both refitting the coefficients and baseline hazard) versus recalibrating the baseline hazard only, we compared fully refitted landmark models at each landmark time with a model version that holds the coefficients from the first landmark (LM1) constant but updates only the landmark-specific baseline hazard S₀(t) and covariate values. Discrimination and calibration were virtually identical between the two approaches (**Tables G, H, and I in** **S1 Table**), and the strength and direction of each predictor’s association with ESRD risk remained remarkably stable across landmarks (**Fig 3A; Table L in** **S1 Table**). These findings demonstrate that shifts in absolute risk over time are driven predominantly by changes in the baseline hazard and covariate measures, reflecting aging, cohort attrition, and evolving clinical profiles, rather than by alterations in covariate effects. This insight underscores the efficiency of landmarking by simply recalibrating S₀(t), and shows we can maintain high predictive accuracy over extended horizons with minimal computational overhead. However, one should note that this finding is study-specific and may not be generalizable to other outcomes and cohorts.

Embedding landmark-based risk updates into the EHR can transform renal care in several ways. First, automated risk recalculation at routine visits could flag patients whose trajectories are accelerating, prompting timely initiation or intensification of renoprotective therapies [20]. Second, health systems can use dynamic estimates to prioritize nephrology referrals and allocate care management resources efficiently. Third, sharing evolving risk profiles with patients enhances engagement and supports shared decision-making around lifestyle and medication adherence. Finally, because predictor coefficients remain stable, simple recalibration of the baseline hazard is sufficient to maintain long-term accuracy, striking a practical balance between methodological rigor and operational feasibility.

The translational impact of the ESRD-DRS score depends on thoughtful implementation in EHR systems. A key advantage of the dynamic landmark framework is that clinicians can view not only a patient’s current predicted risk but also the risk trajectory across successive landmark evaluations. An accelerating trajectory provides an intuitive signal to intensify renoprotective therapy or initiate nephrology referral, whereas a stable or declining trajectory confirms effective management. Individual-level SHAP values identifying the top contributing factors for each patient's risk estimate can be displayed alongside the score to support clinical interpretation. The score can be flexibly dichotomized using empirically selected thresholds (e.g., **Table M in** **S1 Table**) to define categorical risk tiers for automated alerts, with higher-specificity thresholds preferred for triggering clinical workflows. The ESRD-DRS complements existing CKD staging and KFRE by filling an upstream niche: quantifying progression risk at the time of diabetes diagnosis, before established CKD criteria are met. Our framework requires population-level imputation and recalibration, and thus scores must be calculated for all patients in a given health system simultaneously and refreshed at a fixed frequency. Monthly or quarterly batch updates would be operationally feasible, following the precedent of the VA’s Care Assessment Needs (CAN) score [21], which is calculated from structured EHR data, updated at regular intervals, and displayed on clinician dashboards. For patient-facing applications, simplified risk trajectories could be integrated into patient portals to support shared decision-making.

Key strengths of our work include large, diverse development and validation cohorts; explicit competing-risk modeling; high-dimensional EHR data integration; and head-to-head comparison of refitting versus recalibration in dynamic prediction via landmarking. Limitations stem from the use of retrospective EHR data, which may misclassify ESRD events, particularly for veterans receiving care outside the VA, and from residual missingness in biomarkers despite our mitigation strategies. In particular, UACR had > 80% missingness and required ad hoc imputation of a median healthy value (7.5 mg/g) for RECODe and KFRE comparison; because UACR is a key predictor in these equations, its high level of missingness may differentially bias head-to-head comparisons. A missingness indicator was included in ESRD-DRS to partially address this, recognizing that missingness itself may be informative. We are also unable to account for patients who transition to dialysis outside the VA when defining ESRD outcomes in VA EHRs. Moreover, AoU’s lower reported mortality suggests possible under-reporting. Prospective evaluation in routine clinical settings is needed to confirm real-world utility and impact on patient outcomes.

In conclusion, our landmark-based, dynamic ESRD risk model, developed in 708,435 Veterans with newly diagnosed diabetes and externally validated in 13,223 All of Us participants, offers a scalable, clinically feasible approach for long-term risk stratification. ESRD-DRS achieved AUROCs of 0.93, 0.90, and 0.85 for 1-, 5-, and 10-year ESRD risk at landmark year 1, with comparable performance in external validation (AUROCs 0.94, 0.91, and 0.86). Across cohorts, landmark periods, and time horizons, ESRD-DRS outperformed RECODe and KFRE in both discrimination and calibration. The effects of key predictors, including eGFR, BUN, proteinuria, and age, were stable across landmark years, and periodic recalibration of the baseline hazard alone preserved performance over decades, though fully dynamic updating of coefficients may be warranted in conditions with more rapidly shifting risk factors or emerging biomarkers. Future work should assess the model's impact on clinical decision-making, patients’ outcomes, and cost-effectiveness when deployed in real-time EHR systems.

## Materials and methods

### Ethics statement

This study was reviewed and approved by the Institutional Review Board of the Phoenix VA Health Care System, Phoenix, AZ (IRB protocol #1700097–20, “Dynamic Prediction of Short-Term and Long-Term Diabetes Complications Leveraging Massive Electronic Health Records, Million Veteran Project, Machine Learning/Artificial Intelligence, and High-Performance Computing”). For analyses based on Veterans Health Administration electronic health records, the IRB granted a waiver of written informed consent because the research involved secondary use of existing data, posed minimal risk to participants, and all data were de-identified prior to analysis. For the Million Veteran Program component, all participants provided written informed consent at the time of enrollment. All procedures were conducted in accordance with the Declaration of Helsinki and applicable VA and federal regulations. Analyses using data from the NIH All of Us Research Program were conducted under protocols approved by the All of Us Institutional Review Board, and all All of Us participants provided written informed consent at enrollment. All procedures adhered to the principles of the Declaration of Helsinki and applicable VA, NIH, and U.S. federal regulations.

### Study design and cohort assembly in Veterans Health Administration

We used VHA’s longitudinal EHRs to build training and internal testing cohorts (**Fig A in** **S1 Fig**):

(1) Index date: first diabetes (type 1 or 2, per modified Klompas algorithm [22]) diagnosis date. It was defined as the earlier of (a) a diabetes ICD code or (b) a prescription for insulin or any non-metformin glucose-lowering drug.
(2) Pre-index requirements: ≥ 1 outpatient visit (without a diabetes diagnosis) in each of the two years before the index date, to exclude pre-existing diabetes, and ≥ 1 post-index low-density lipoprotein (LDL), HbA1c, and creatinine measurement. Patients missing key demographic variables (i.e., date of birth and gender) were excluded.
(3) Data extracted: From the index date until the outcome or censoring, we extracted demographics, longitudinal diagnoses and CPT (Current Procedural Terminology) codes, medication use, laboratory values, and health factors (e.g., smoking; **Fig 1A**).

### Outcome definitions in VHA

Composite ESRD was defined as:

-  ≥ 2 EHR entries (diagnosis or procedure) for ESRD, i.e., stage 5 + CKD, dialysis, or kidney transplant, 45–365 days apart; **or**
-  ≥ 2 consecutive eGFR measures < 15 mL/min, ≥ 45 days apart.

Time-to-event was measured from the index date to the first ESRD or death; those lost to follow-up were censored at their last visit. Death (the competing risk) was ascertained via aggregated Social Security Administration, Centers for Medicare & Medicaid Services, VHA, VA National Cemetery records, and Corporate Data Warehouse death data, with 98.3% sensitivity and 97.6% date agreement vs. National Death Index; **Fig 1B**.

### Feature engineering and processing in VHA EHRs

More than 400 variables were extracted and processed from EHRs for model training. *Laboratory results*. The laboratory results were defined and captured using the Million Veteran Program (MVP) Core Data for 53 common laboratory tests [23–25] (**Table A in** **S1 Table**) [24,25]. *Health Conditions and Procedures.* International Classification of Diseases (ICD) diagnosis codes were processed to define features, outcomes, and Diabetes Complications Severity Index (DCSI) score [24–26] (detailed in **Table C in** **S1 Table**). Additional variables were determined using previously published algorithms or by manual inspection of ICD codes (**S1** **Methods**). *Medications*. Medications were defined per prior work [24,25], with additional drugs identified by pattern-matching fields (e.g., “DrugNameWithoutDose,” “VADrugClassification”) and manual review (**Table D in** **S1 Table**). For each time window, we flagged a drug as present if ≥1 prescription appeared; duration was taken from days-supply, with any supply <30 days rounded up to a 30-day exposure. *Variable processing*. Baseline features were captured at LM1 (one-year post-diagnosis) using windows of 2 years pre-diagnosis for biomarkers, 30 days for medications, and all available years for comorbidities, diagnoses, and smoking. When multiple observations existed, the last pre-LM1 value was used. Continuous predictors with ≤40% missingness (**Table A in** **S1 Table**) were multiply imputed using the ***mice*** R package separately for the training and validation datasets (see **S1 Methods**), and one imputation replicate was used for downstream analysis. Predictors with >40% missingness were binned by clinical cutoffs, with missingness as its own category. All categories and continuous (imputed) markers were included as potential predictors.

### Dynamic prediction of ESRD with competing risks by landmarking

Dynamic landmarking was applied at 1-, 5-, and 10-year post-diabetes diagnosis (LM1, LM5, LM10) [27,28]. At each landmark time, the training and testing sets were updated to include the most recent feature values up to that point (**Fig 1A**), and models were evaluated at 1-, 5-, and 10-year horizons (H1, H5, H10). The LM1 cohort was split 70/30 into training and testing sets, and these same splits were retained for LM5 and LM10.

We modeled ESRD risk via an FG SDH approach with all-cause mortality as a competing event. Predictor selection was two-stage:

1. **Univariable screening:** age-, sex-, and race-adjusted logistic regressions; retain predictors with |logOR| > 0.1 and P < 0.05.
2. **Penalized FG SDH:** apply an MCP for computational efficiency and sparsity. MCP tuning maximized the 10-year area under the time-dependent receiver operating characteristic curve (AUROC) via 5-fold cross-validation.

Post-selection, unpenalized FG SDH models were re-fit at LM5 and LM10 using the same predictors. [29].

We quantified the model’s discrimination by reporting the area under the time-dependent receiver operating characteristic (ROC) curve (AUROC) and the area under the precision (i.e., positive predictive value)- recall (i.e., sensitivity) curve (AUPRC). We assessed the model’s calibration performance using the Brier score, calibration plots, and calibration slopes derived from pseudo-observations [30–32]. Calibration plots were constructed by stratifying patients into deciles (VHA) or quartiles (AoU) of predicted risk, and slopes were estimated by regressing pseudo-observations on predicted risk; a slope of 1 indicates perfect calibration [32,33].

### Recalibration

When there is a systematic over- or underestimation of risk, transporting a prediction function from one setting to another or over time requires recalibration [7] (**S1 Methods**). For each validation, model coefficients were exported to the target cohort. However, predicted probabilities of the event were recalibrated using the target cohort’s own incidence rate, estimated using Cox or the FG SDH model, at the mean value of the predictors.

### Subgroups

We evaluated our models in several key subgroups: stratified by race, ethnicity, sex, age at diabetes diagnosis (over or under 65, to assess sensitivity to use of Medicare), decade of diabetes diagnosis (2000s vs. 2010s, to determine influence of newer medications), and baseline eGFR (≥60 vs. < 60 mL/min/1.73 m², to assess sensitivity to baseline renal function).

### Variable importance

Variable importance was assessed using SHAP (SHapley Additive exPlanations) value [33], which quantifies how each top predictor shifts an individual’s estimated risk relative to a baseline. Aggregated SHAP values yield a global ranking of feature importance, while individual-level values expose patient-specific variation in feature effects. For comparison, we also extracted and examined the FG SDH model’s coefficients.

The R package “fastcmprsk” [34], tailored for fitting large FG SDH models, was used to generate predictions. “timeROC” [35] was employed for model evaluation, and the SHAP values were calculated using “fastshap” [33].

### External validation in the AoU cohort

In AoU, we identified a cohort of newly diagnosed diabetes patients using the same criteria as applied in VHA (**Fig B in** **S1 Fig**). Patients who reported using military health insurance were excluded to avoid overlap with the VHA population. Conditions and procedures were defined using the same codes and patterns as those used in the VHA. Biomarkers were similarly defined (**Table E in** **S1 Table**). Medications were defined using OMOP concept sets, and resulting records were reviewed to confirm relevance and route of administration (**Table D in** **S1 Table**). Subgroup selection, variable transformation, categorization, censoring, and multiple imputation were conducted in the same manner as in VHA.

Model coefficients trained in VHA were exported to AoU to generate risk scores for ESRD in Controlled Tier Dataset v8 of AoU [workspace aou-rw-96deadda]. We evaluated all model discrimination and calibration metrics across landmark times and evaluation horizons for the overall cohort and subgroups. The selected predictors from ESRD-DRS were used to fit an unpenalized FG SDH model at AOU LM1, and the effect size estimates from this model were compared to those obtained in VHA LM1.

### Benchmark with RECODe and KFRE risk scores

We generated and evaluated risk scores based on the RECODe risk equation for ESRD [14]. RECODe risk equations were developed among T2D patients from the Action to Control Cardiovascular Risk in Diabetes study (ACCORD) and used clinically adjudicated ESRD events. We created RECODe scores for all 3 landmark periods in both VHA and AoU. As RECODe requires a continuous UACR value, and UACR had > 80% missing rate, patients without any measurement of UACR in the landmark period were assigned a value of 7.5 mg/g (the median healthy (<30mg/g) UACR value in the cohort). Model discrimination and calibration performance were then evaluated as above in both cohorts, for both the overall cohort and subgroups. We similarly calculated and evaluated the 4-variable KFRE score [36](**S1 Methods**).

### Optimal thresholds for clinical decision-making

We evaluated optimal probability thresholds for classifying risk using predicted probabilities from our ESRD-DRS model within the VHA. Thresholds were derived using two commonly applied criteria: Youden’s J statistic (maximizing sensitivity + specificity − 1) [37] and the Top-Left (minimum distance) method (minimizing the Euclidean distance to the point [0,1] on the ROC curve) [38]. For each approach, we calculated the corresponding sensitivity, specificity, and threshold values.

## Supporting information

S1 Methods Supplementary methods. (DOCX)

S1 Table Supplementary tables. (XLSX)

S1 Fig Flowchart of VHA and AoU diabetes patients’ cohort generation. (DOCX)

S2 Fig Cumulative incidence function ESRD and Death in VHA and in select subpopulations across different landmark times. (DOCX)

S3 Fig Distribution of biomarker measures extracted from VHA, AoU, separated by sex. (DOCX)

S4 Fig Distribution of the number of repeated measurements of biomarkers extracted from VHA, AoU, separated by sex. (DOCX)

S5 Fig Calibration plots at three landmark times and for three horizons in VHA. (DOCX)

S6 Fig Calibration plots at three landmark times and for three horizons in AoU. (DOCX)

S7 Fig Calibration plots at three landmark times and for three horizons among select VHA subpopulations. (DOCX)

S8 Fig AUROC of dynamic prediction, KFRE, and RECODe models for ESRD in VHA among select subpopulations. (DOCX)

S9 Fig SHAP values for the top 10 features among patients with ESRD, across three landmark models and three horizon times. (DOCX)

S10 Fig SHAP values for top 10 features in VHA across three landmark models and three horizon times. (DOCX)
