# Supplementary material for: Dynamically predicting renal failure after development of diabetes across biobanks
Source: PLOS Digit Health. 2026 May 4;5(5):e0001375. doi: 10.1371/journal.pdig.0001375 (PMC13138643; doi:10.1371/journal.pdig.0001375)
Supplement: S1 Methods — (DOCX) [file pdig.0001375.s001.docx]

**Supplementary Methods**

Table of Contents

[Phenotyping – VHA 4](#_Toc224894386)

[SQL Data Pull 4](#_Toc224894387)

[Diabetes 4](#_Toc224894388)

[Demographics 4](#_Toc224894389)

[Smoking 5](#_Toc224894390)

[End-Stage Renal Disease 5](#_Toc224894391)

[Death 5](#_Toc224894392)

[Biomarker Extraction and Cleaning 5](#_Toc224894393)

[Conditions 6](#_Toc224894394)

[Procedures 6](#_Toc224894395)

[Medications 7](#_Toc224894396)

[RECODe and KFRE 7](#_Toc224894397)

[Phenotyping – All of Us (AoU) 9](#_Toc224894398)

[SQL Data Pull 9](#_Toc224894399)

[Diabetes 9](#_Toc224894400)

[Insurance 9](#_Toc224894401)

[Race/Ethnicity 9](#_Toc224894402)

[Smoking 9](#_Toc224894403)

[End-Stage Renal Disease 9](#_Toc224894404)

[Death 10](#_Toc224894405)

[Biomarker Extraction and Cleaning 10](#_Toc224894406)

[Conditions 10](#_Toc224894407)

[Procedures 10](#_Toc224894408)

[Medications 10](#_Toc224894409)

[Analyses 12](#_Toc224894410)

[Defining Time Intervals 12](#_Toc224894411)

[Landmarking 12](#_Toc224894412)

[Imputation 12](#_Toc224894413)

[Model Fitting – Landmark 1 12](#_Toc224894414)

[Model Fitting – Landmarks (LM) 5 and 10 13](#_Toc224894415)

[Subgroup Analysis 13](#_Toc224894416)

[AUCROC 13](#_Toc224894417)

[AUPRC 13](#_Toc224894418)

[Refitted Models, Calibration, and Recalibration 14](#_Toc224894419)

[SHAP 14](#_Toc224894420)

# **Phenotyping – VHA**

**SQL Data Pull**

All data were extracted from the Veterans Health Administration (VHA) database, with a pull date of March 6, 2023. Data included demographic information, diagnoses, procedures, medications, laboratory results, vital signs, health factors, visit dates and types, and mortality records.

**Diabetes**

Patients were categorized into Type 1 Diabetes (T1D), Type 2 Diabetes (T2D), or unspecified diabetes based on diagnosis codes and medication records. Diagnosis codes were extracted using the patterns “^250|^362.0[1-7]|^366.41|^E1[013]|^E08|^O24[0138]”. Codes were then categorized as indicating T1D or T2D:

- T2D: Diagnosis codes containing the patterns "type II|type 2" (case insensitive).
- T1D: Diagnosis codes containing the patterns "type I|type 1|juvenile" (case insensitive), but not the patterns associated with T2D.
- Unspecified Diabetes: Codes not matching the patterns for T1D or T2D remained uncategorized.

A refined classification approach was used to differentiate between T1D and T2D. The classification algorithm followed a modified version of the Klompas algorithm, which did not consider urine acetone strips [1]. The following criteria were applied:

- Patients were assigned to T1D if more than 50% of their diabetes-related diagnostic codes were T1D codes **AND** used insulin at any point **AND** they **EITHER** had a documented record of glucagon use **OR** had no records of oral glucose-lowering medications (except metformin).
- Patients were assigned to T2D if they did not meet T1D criteria **AND** had at least two T2D diagnosis codes recorded at least 30 days apart.

## **Demographics**

- Race and ethnicity were assigned based on the mode self-reported value.
- Marital status categories of "divorced" and "separated" were combined into a single category. “Single” and “never married” were combined into a single category.
- Unknown marital status was retained as a separate category rather than excluding patients with missing values.
- Patients were filtered to those born in 1938 or later due to a large quantity of missing dates of death for patients born before this date, indicating an implausibly high rate of survival.

**Smoking**

Smoking status was determined from structured electronic health record (EHR) health factors. Categories included "never smoker," "former smoker," and "current smoker" [2]. For another version of this variable, former and current smokers were combined into “ever smokers”.

**End-Stage Renal Disease**

ESRD was identified using ICD-9 and ICD-10 diagnosis codes, as well as estimated glomerular filtration rate (eGFR) derived from laboratory measures of serum creatinine. Patients were considered to have ESRD if:

- Diagnosis or procedure codes for ESRD, Stage 5 kidney disease, dialysis, or kidney transplant were present on at least 2 separate dates between 30 and 365 days apart.
- eGFR was less than 15 for a period of at least 45 days, as measured by 2 or more consecutive serum creatinine lab results.

**Death**

Death was ascertained using aggregated death data from the Social Security Administration, Medicare and Medicaid Services, VHA death benefits, VHA hospitals, and VA National Cemetery Association records [Refer to Acknowledgment for Reference]. Patients with medical records appearing more than 60 days after a recorded date of death were excluded. The proportion of patients recorded as deceased was analyzed across year of birth to ensure consistency and reliability of death reporting.

## **Biomarker Extraction and Cleaning**

- Laboratory test values were mapped using adjudicated definitions from the Million Veteran Program (MVP), which matched database values based on LabChemTestName, sta3n, and Topography. The mapping was extracted from MVP in 2018. We included only mappings that had a consensus among MVP adjudicators, unless otherwise noted.
- The most common units were identified for each biomarker, with unreasonable units filtered out. Unit conversions were performed where possible, and converted values were compared against original values to ensure consistency.
- Additional quality control checks were applied to specific biomarkers. Notably:
  - **LDL Cholesterol (LDLC):** Included laboratory results with LOINC codes 2089-1, 13457-7, and 18262-6, even in cases where adjudicator consensus was not reached.
  - **Potassium:** Excluded laboratory tests with LabChemTestName patterns containing "PO4" or "PROTEIN."
  - **HDL Cholesterol (HDLC):** Included laboratory results with LOINC code 2085-9 even in cases where adjudicator consensus was not reached. Excluded laboratory tests with LabChemTestName patterns containing "LDL", "NON-HDL", or “NON HDL”.
  - **Uric Acid**: Did not require consensus among MVP adjudicators. Filtered topographies to serum, plasma, or blood.
  - **CCP**: Did not require consensus among MVP adjudicators. Those LabChemTestName without a consensus were required to contain the strings “cyclic citrull” or “ccp” (case insensitive). Filtered topographies to serum, plasma, or blood.
  - **Total Bilirubin**: Did not require consensus among MVP adjudicators. Filtered topographies to serum, plasma, or blood.
  - **Direct Bilirubin**: Did not require consensus among MVP adjudicators. Filtered topographies to serum, plasma, or blood.
  - **Bilirubin Urine Stick**: Did not require consensus among MVP adjudicators. Filtered topographies to urine. Values were mapped according to the level of severity (0-3, with 0 being negative and 3 being the highest). Values were mapped in the following order according to matched patterns: “Mod|2+” = 2, “Large|LG|3+” = 3, “Neg|^N$” = 0, “Sm|1+|POS|TR” = 1, otherwise NA.
  - **Amylase**: Extracted using LOINC code 1798-8.
  - **GGTP**: Extracted using LOINC code 2324-2.
  - **Total Protein**: Extracted using LOINC code 2885-2.
  - **BMI** was calculated at each measurement of weight using the patient’s median recorded height across all measurements.
  - **eGFR** was calculated from serum creatinine using the CKD EPI 2021 equation [3].
- Measurements were filtered to an allowable range, determined using the CIPHER resource (when available), or manual inspection coupled with expert review.

**Conditions**

Medical conditions were identified using ICD-9 and ICD-10 diagnosis codes obtained from fee-for-service, inpatient, and outpatient records. ICD-9 and ICD-10 codes were merged, with letters for ICD-9 codes (used before October 1, 2015) changed to lowercase, and all search terms required to match from the first character in the field. Most features were defined using previously described algorithms [4, 5]. Additional variables were defined for diabetic retinopathy [6], urolithiasis, Lupus, Sleep Disorders, Nephrotic Syndrome, Focal Segmental Glomerulosclerosis (FSGS), Autosomal Dominant Polycystic Kidney Disease (ADPKD), Cancer[7], and diagnoses of proteinuria, using previously published ICD code lists or by manual inspection and review of ICD codes. ICD codes were also used to generate the time-dependent Diabetes Complication Severity Index (DCSI) score [8] and Elixhauser score [9, 10].

**Procedures**

Procedures were extracted from the database using CPT and ICD procedure codes using previously described algorithms [4]. Data sources included inpatient, outpatient and fee for service.

## **Medications**

The drugs and drug categories for antipsychotics, stimulants, opioid-for-pain, estradiol, levothyroxine, ezetimibe, bile acid sequestrants (BAS), aspirin, and metformin were defined as previously described[4, 5]. Additional medication variables were defined for subclasses of glucose-lowering medications, fibrates, niacin, anticoagulants, NSAIDs, platelet aggregation inhibitors, proton pump inhibitors, antivirals, protease inhibitors, antibiotics, antineoplastics, and systemic corticosteroids. These variables were defined by searching for relevant patterns in several fields (“DrugNameWithoutDose”, “VADrugClassification”, etc.), and manually inspecting the results to confirm relevance. Details of the extraction procedure are shown in **Table D in S1 Tables.** Medications were recorded as present in each time window of data collection if one or more instances were recorded and absent if none of the medications included for each variable was present.

- Prescription fills were analyzed, including dosage, quantity, and days supply.
- Data sources included inpatient, outpatient, BCMA, and IV
- Prescriptions marked as returned to stock were excluded
- Investigational and placebo medications were excluded by filtering out drug names containing the patterns:
  - "PLACEBO|OR PBO|/PBO|STUDY|TRIAL|COOP #|CSP #|INV-|INV[ :#]|ACCORD|IRB#"
- Medications classified under VA categories beginning with "IN" (investigational), "DX," "PH," "XA," or "AS" were excluded.
- Records missing a local drug name were also excluded.
- For some medications, days supply was imputed using the mode value specific to each dosage/quantity, typically 30 or 90 days. For all medications, medication status (0 or 1) was considered positive (1) for 30 days after the initial fill, or for the Days Supply, whichever was longer.

Statins were mapped into low, medium, and high dosage prescriptions based on the drug name and dosage following Grundy et al [11]. High-intensity statins generally reduce LDL-C levels by 50% or more, while moderate-intensity treatments lower them by 30% to 49%, and low-intensity statins result in less than a 30% reduction. Drug names were captured using the pattern “STATIN” and then systematically mapped onto dosage categories based on observed drug names with a dose.

## **RECODe and KFRE**

We calculated the Risk Equations for Complications of Diabetes kidney disease score (RECODe)[12], which is a predictive model developed to estimate the risk of kidney disease progression in individuals with diabetes.

- Calculated using raw laboratory values before transformation.
- UACR had a high rate of missingness. To address this, we used median imputation using only measurements within the reference range.
- CVD history was defined as a composite of 6 of our existing features; EH_HEARTFAILURE, EH_VALVDIS, EH_PERIVASC, CAD, MI, Stroke_infarct
- BP lowering drugs were defined as a composite of several of our existing features: BetaBlockers, BetaB2nd, BetaB3rd, CChannelBlockers, ACEInhibitors, AlphaBlockers, AngiotensinIIRB, AntiHypertensiveComb, ThiazideDiuretics, PotassiumSparingDiuretics, PeriphVasodilators, LooopDiuretics, Other_Antihypertensives
- Oral diabetes drugs was defined as a composite of several of our existing features: Thazolidinedione, DPP4, SGLT2, Sulfonylurea, Metformin, Other_GlucoseLowering

We also calculated the 4-variable Kidney Failure Risk Equation (KFRE) score[13, 14], a predictive model developed to estimate the risk of End-Stage Renal Disease (ESRD) in the general population.

- This equation includes age, sex, eGFR, and UACR
- UACR values were imputed using the same method used for calculating the RECODe score.

# **Phenotyping – All of Us (AoU)**

**SQL Data Pull**

All data were extracted in February through May 2025 from the AoU Controlled Tier Dataset v8. Data included demographic information, diagnoses, procedures, medications, laboratory results and measurements, visit dates and types, and mortality status.

**Diabetes**

Diabetes classification followed a similar approach to VHA but used Observational Medical Outcomes Partnership (OMOP) concept IDs to extract diabetes codes. Diagnosis codes were extracted from structured health records, and classification followed the modified Klompas algorithm described above.

**Insurance**

Insurance status was determined from survey answers at the time of AoU enrollment. Patients reporting VA or Military health insurance were excluded.

## **Race/Ethnicity**

Race and ethnicity (Hispanic/Latino/Spanish or non-Hispanic) were derived from survey answers at the time of AoU enrollment. We collapsed the categories to match those used in VHA; missing, skipped, refused to answer, “More than one population”, Middle Eastern / North African, and patients answering “None of These” were collapsed into the category of Other/Unknown.

**Smoking**

Smoking status was determined from survey answers at the time of AoU enrollment, and was categorized into "never smoker," "former smoker," and "current smoker".

**End-Stage Renal Disease**

ESRD was defined using the same algorithm used in VHA, but used OMOP concept IDs to extract ESRD codes. Source concept codes were reviewed to ensure the severity of kidney disease was mapped appropriately; records with source concept codes corresponding to kidney disease stages 1-4 were removed. Records were extracted from the Condition, Procedure, and Observation domains. Dialysis records were also extracted from the Device domain. The resulting phenotype was then compared to self-reported kidney transplant status at the time of the baseline survey. Patients who self-reported receiving a kidney transplant, but had no evidence of transplant in their EHRs were excluded.

**Death**

Mortality status was sourced using structured EHR data and from HealthPro reporting.

**Biomarker Extraction and Cleaning**

Most biomarkers extracted in VHA were also extracted in AoU using curated OMOP concept sets. Laboratory results were filtered to the same allowable range as in VHA, and similar QC was applied except where noted below.

- **Urine Bilirubin Test Strips:** Values were categorized into three levels: "normal/absent," "small," "medium," and "large" for labs with range 0-4, 1-4, or 0-3. Values outside these ranges were excluded.

**Conditions**

Conditions (except diabetes and ESRD) were derived from ICD-9/ICD-10 codes. Variable definitions (code lists/patterns) were the same across VHA and AoU. ICD-9 and ICD-10 codes were matched from the “source_concept_code” field of the “condition_occurrence” OMOP table.

**Procedures**

Most procedures (except renal transplant, which also used ICD diagnosis codes, and dialysis, which also used ICD diagnosis codes and device codes) were extracted from CPT and ICD procedure codes matched from the “source_concept_code” field of the “procedure” OMOP table. Variable definitions were the same across VHA and AoU.

**Medications**

Medication variables were defined using OMOP concept sets, which included all concept descendants. For each medication, records were processed similarly to VHA. No attempt was made to exclude investigational/placebo drugs. Medication concept names were processed to exclude dosing/brand information; each generic name was inspected to confirm relevance to the relevant medication class/variable. For some medication classes (i.e. blood pressure, lipid-lowering, and diabetes medications), sub-classes (e.g. ACE Inhibitors, Bile Acid Sequestrants, Sulfonylureas) were distinguished using pattern matching. Statins were divided into high-dose, medium-dose, and low-dose prescriptions consistent with the analysis in VHA.

# **Analyses**

**Defining Time Intervals**

Time-dependent variables were defined for longitudinal analysis using the survival R package. All clinical variables were aligned to the date of first diabetes; times were originally expressed in years since diabetes diagnosis and rounded to the nearest 0.1 year.

## **Landmarking**

Snapshots of all patients’ clinical features and outcome status were collected at 1.0, 5.0, and 10.0 years after their first diabetes diagnosis. At each of these landmarks, patients who had experienced ESRD, death, or loss to followup were removed, and time-to-event was redefined as years since the landmark time.

## **Imputation**

In VHA, we imputed the missing values for biomarker variables with a rate of missingness of <= 40% at this time point using the ***mice*** R package (v3.16.0)[15], then log transformed triglycerides, WBC, and platelet count, then standardized all imputed continuous variables. All predictors with correlation coefficients of 0.05 or greater and a minimum proportion of usable cases of 1% (for categorical variables) were used for the imputation of each variable. Biomarker variables with missing rates > 40% were categorized based on meaningful clinical cutoffs (**Table E in S1 Tables**), with missing values being coded as a category of their own and included in the datasets as potential predictors. Biomarker variables with a missingness rate of >40% were dropped for modeling purposes, while the categorized versions of these variables were retained. Smoking status, Race, and Ethnicity were not imputed; missingness of these variables was treated as a category of its own. Imputed values were carried forward to future time points until an updated value was observed. Five multiple imputation datasets were generated. All 5 multiple imputation datasets were compared to ensure a similar distribution of imputed values. Empirical comparisons show similar prediction performance across imputation replicates. Therefore, only the first replicate was used for subsequent analysis. Code for multiple imputation is provided at

https://github.com/cpm-lab/ESRD-DRS/blob/main/KDI/3.analysis_scripts/3.impute_t1.R.

In AoU, we performed imputation for the same variables that were imputed in VHA, regardless of the rate of missingness in AoU. Categorical variables were retained to match those retained in VHA. Certain continuous biomarker variables (BMI, Pain) were supplemented using baseline survey data prior to imputation. Imputation was then performed in the same manner as in VHA.

## **Model Fitting – Landmark 1**

We fit Fine-Gray sub-distribution hazard (FG SDH) competing risk models for ESRD with all-cause mortality as the competing risk using the *fastcmprsk* R package[16]. Feature selection consisted of 2 steps: (1) Simple logistic regression models were fit separately for each feature, with age, gender, and race as covariates. Features with absolute effect sizes (i.e., log of odds ratio) greater than 0.1 (i.e., |logOR| > 0.1) and P values < 0.05 were considered for the next step. (2) The minimax concave penalty (MCP) was chosen as the regulation and feature selection method due to the computational efficiency and the balance between model performance and complexity (i.e., the fewer variables selected). We tuned the tuning parameter based on the 10-year area under the Receiver-Operating Characteristic curve (ROC) curve (AUC) using a 5-fold cross-validation (CV) within the training set to control overfitting.

## **Model Fitting – Landmarks (LM) 5 and 10**

To ensure a consistent variable list across LM times, the variable selection was only performed at LM1. After variable selection, unpenalized models were re-fit at LM5 and LM10 using the same set of predictors.

## **Subgroup Analysis**

Patients were subset by certain variables of interest to evaluate model performance and calibration in these subgroups, in addition to the overall cohort. These variables included age (over vs. under 65 years), sex (Male and Female), race (White/Caucasian and Black/African American), ethnicity (Hispanic/Latino and Not Hispanic/Latino), decade in which diabetes was first diagnosed (2000s and 2010s), and eGFR value at the relevant landmark time (over 60 and under 60 ml/min/m^2^). Not all categories of race, ethnicity, and decade of diabetes diagnosis were included, and not all subgroups were mutually exclusive.

## **AUCROC**

At all landmarks, cause-specific time-dependent AUC was calculated using the timeROC R package[17]. We report the performance at three horizon times (1, 5, and 10 years after each landmark). For each landmark, we calculated a 95% confidence interval for the AUROC using 500 bootstrap samples (1000 in AoU analyses due to the smaller sample size and low event counts).

## **AUPRC**

At all landmarks, cause-specific time-dependent precision and recall was calculated using the timeROC R package. Area under the precision-recall curve (AUPRC) was then estimated using the pracma R package. We report the AUPRC at three horizon times (1, 5, and 10 years after each landmark) along with the cumulative incidence at these times, which is necessary for interpreting the AUPRC. Confidence intervals were not reported due to computational time limits.

## **Refitted Models, Calibration, and Recalibration**

**Refitted Models**. To assess the relative importance of updating feature values vs. updating model coefficients in dynamic prediction, the minimax concave penalty penalized model fitted at LM1 was directly used to evaluate three horizon times for LM5 and LM10, and was compared to the refitted unpenalized models at LM5 and LM10. For both models, the models were recalibrated at different LM times (see below).

**Recalibration**. When evaluating the calibration of a score (trained in a source cohort) in an internal or external validation cohort (i.e. ESRD-DRS (trained in VHA) and RECODe (trained in ACCORD) in AoU, RECODe (trained in ACCORD) in VHA), we first generated the appropriate risk score using the coefficients trained in the source cohort and the target cohort’s covariate values. Then we generated predicted probabilities of ESRD while recalibrating the model for the target cohort’s cumulative incidence of the event at the mean values of the target cohort’s covariates. First, we used fastcmprsk::fastCrr to fit a model in the target cohort using the risk score as the only covariate. By doing so, we estimate updated Breslow jumps at each time interval, which, together with the risk score derived from the appropriate coefficients, can be used to predict the recalibrated probability of the event. For each landmark and horizon, we calculated the time-specific probability of the event at the **mean value of the risk score** (S_0_(t)) using fastcmprsk::predict.fcrr(). Then, we calculated the probability of the event for all patients using the formula:

P(t) = 1 - S_0_(t)^exp(RskScore – mean(RiskScore))^

Recalibration was performed at the cohort level and was not performed within subgroup analyses.

**Calibration evaluation.** We assessed how closely predicted outcomes matched actual outcomes using Brier scores, calibration plots, and the calibration slope derived from jackknife pseudo-observations [21-23]. The Brier score is the mean squared difference between predicted risks and observed events. For calibration plots, individuals were grouped into deciles (in VHA) or quantiles (in AoU) based on their predicted risk; the observed outcome proportions in each group were then plotted against the mean predicted risk for a specified horizon year. Calibration plots with a linear smoother were also generated. A calibration slope is derived via linear regression with pseudo-observations [24, 25] as the outcome and an intercept of 0. A slope of 1 indicates optimal calibration.

**SHAP**

SHapley Additive exPlanations (SHAP) values were computed to quantify the contribution of individual features to predicted outcomes for each patient using the fastshap R package[18], using 15 Monte Carlo repetitions for estimating each Shapley value.

Reference

1. Schroeder EB, Donahoo WT, Goodrich GK, Raebel MA. Validation of an algorithm for identifying type 1 diabetes in adults based on electronic health record data. Pharmacoepidemiol Drug Saf. 2018;27(10):1053-9. Epub 20180102. doi: 10.1002/pds.4377. PubMed PMID: 29292555; PubMed Central PMCID: PMCPMC6028322.

2. Golden SE, Hooker ER, Shull S, Howard M, Crothers K, Thompson RF, et al. Validity of Veterans Health Administration structured data to determine accurate smoking status. Health Informatics J. 2020;26(3):1507-15. Epub 20191107. doi: 10.1177/1460458219882259. PubMed PMID: 31697173.

3. Inker LA, Eneanya ND, Coresh J, Tighiouart H, Wang D, Sang Y, et al. New Creatinine- and Cystatin C-Based Equations to Estimate GFR without Race. N Engl J Med. 2021;385(19):1737-49. Epub 20210923. doi: 10.1056/NEJMoa2102953. PubMed PMID: 34554658; PubMed Central PMCID: PMCPMC8822996.

4. Dhaubhadel S, Kolade B, Ribeiro RM, Ganguly K, Hengartner NW, Bhattacharya T, et al. MACE prediction using high-dimensional machine learning and mechanistic interpretation: A longitudinal cohort study in US veterans. medRxiv. 2022. doi: 10.1101/2022.10.31.22281742.

5. Dhaubhadel S, Ganguly K, Ribeiro RM, Cohn JD, Hyman JM, Hengartner NW, et al. High dimensional predictions of suicide risk in 4.2 million US Veterans using ensemble transfer learning. Sci Rep. 2024;14(1):1793. Epub 20240120. doi: 10.1038/s41598-024-51762-9. PubMed PMID: 38245528; PubMed Central PMCID: PMCPMC10799879.

6. Breeyear JH, Mitchell SL, Nealon CL, Hellwege JN, Charest B, Khakharia A, et al. Development of electronic health record based algorithms to identify individuals with diabetic retinopathy. J Am Med Inform Assoc. 2024;31(11):2560-70. doi: 10.1093/jamia/ocae213. PubMed PMID: 39158361; PubMed Central PMCID: PMCPMC11491608.

7. Hung AM, Assimon VA, Chen HC, Yu Z, Vlasschaert C, Triozzi JL, et al. Genetic Inhibition of APOL1 Pore-Forming Function Prevents APOL1-Mediated Kidney Disease. J Am Soc Nephrol. 2023;34(11):1889-99. Epub 20231006. doi: 10.1681/ASN.0000000000000219. PubMed PMID: 37798822; PubMed Central PMCID: PMCPMC10631602.

8. Young BA, Lin E, Von Korff M, Simon G, Ciechanowski P, Ludman EJ, et al. Diabetes complications severity index and risk of mortality, hospitalization, and healthcare utilization. Am J Manag Care. 2008;14(1):15-23. PubMed PMID: 18197741; PubMed Central PMCID: PMCPMC3810070.

9. Elixhauser A, Steiner C, Harris DR, Coffey RM. Comorbidity measures for use with administrative data. Med Care. 1998;36(1):8-27. doi: 10.1097/00005650-199801000-00004. PubMed PMID: 9431328.

10. Chu YT, Ng YY, Wu SC. Comparison of different comorbidity measures for use with administrative data in predicting short- and long-term mortality. BMC Health Serv Res. 2010;10:140. Epub 20100527. doi: 10.1186/1472-6963-10-140. PubMed PMID: 20507593; PubMed Central PMCID: PMCPMC2897792.

11. Grundy SM, Stone NJ, Bailey AL, Beam C, Birtcher KK, Blumenthal RS, et al. 2018 AHA/ACC/AACVPR/AAPA/ABC/ACPM/ADA/AGS/APhA/ASPC/NLA/PCNA Guideline on the Management of Blood Cholesterol: A Report of the American College of Cardiology/American Heart Association Task Force on Clinical Practice Guidelines. Circulation. 2019;139(25):e1082-e143. Epub 20181110. doi: 10.1161/CIR.0000000000000625. PubMed PMID: 30586774; PubMed Central PMCID: PMCPMC7403606.

12. Basu S, Sussman JB, Berkowitz SA, Hayward RA, Yudkin JS. Development and validation of Risk Equations for Complications Of type 2 Diabetes (RECODe) using individual participant data from randomised trials. Lancet Diabetes Endocrinol. 2017;5(10):788-98. Epub 20170810. doi: 10.1016/S2213-8587(17)30221-8. PubMed PMID: 28803840; PubMed Central PMCID: PMCPMC5769867.

13. Tangri N, Grams ME, Levey AS, Coresh J, Appel LJ, Astor BC, et al. Multinational Assessment of Accuracy of Equations for Predicting Risk of Kidney Failure: A Meta-analysis. JAMA. 2016;315(2):164-74. doi: 10.1001/jama.2015.18202. PubMed PMID: 26757465; PubMed Central PMCID: PMCPMC4752167.

14. Tangri N, Stevens LA, Griffith J, Tighiouart H, Djurdjev O, Naimark D, et al. A predictive model for progression of chronic kidney disease to kidney failure. JAMA. 2011;305(15):1553-9. Epub 20110411. doi: 10.1001/jama.2011.451. PubMed PMID: 21482743.

15. Buuren Sv, Groothuis-Oudshoorn K. mice: Multivariate Imputation by Chained Equations inR. Journal of Statistical Software. 2011;45(3). doi: 10.18637/jss.v045.i03.

16. Kawaguchi ES, Shen JI, Li G, Suchard MA. A Fast and Scalable Implementation Method for Competing Risks Data with the R Package fastcmprsk. The R Journal. 2020;12(2). doi: 10.32614/rj-2021-010.

17. Blanche P, Dartigues JF, Jacqmin-Gadda H. Estimating and comparing time-dependent areas under receiver operating characteristic curves for censored event times with competing risks. Stat Med. 2013;32(30):5381-97. Epub 20130912. doi: 10.1002/sim.5958. PubMed PMID: 24027076.

18. Greenwell B. fastshap: Fast Approximate Shapley Values. 2024.
