# Supplementary material for: Dynamically predicting renal failure after development of diabetes across biobanks
Source: PLOS Digit Health. 2026 May 4;5(5):e0001375. doi: 10.1371/journal.pdig.0001375 (PMC13138643; doi:10.1371/journal.pdig.0001375)
Supplement: S1 Fig — (DOCX) [file pdig.0001375.s003.docx]

# **S1 Fig.**

Flowchart of **A.** VHA and **B.** AoU diabetes patients’ cohort generation.

**A.** **B**.


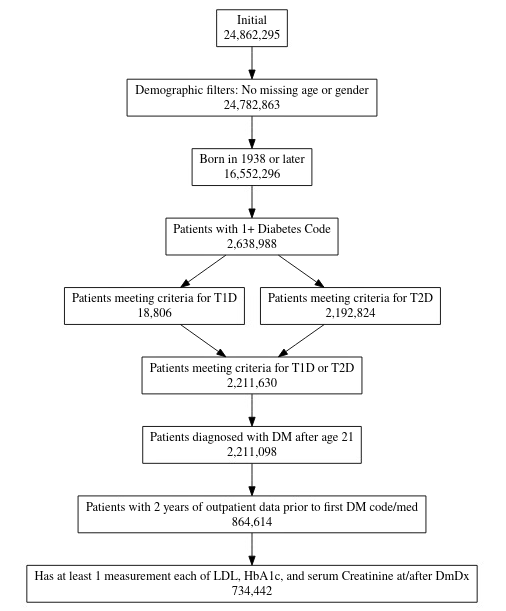

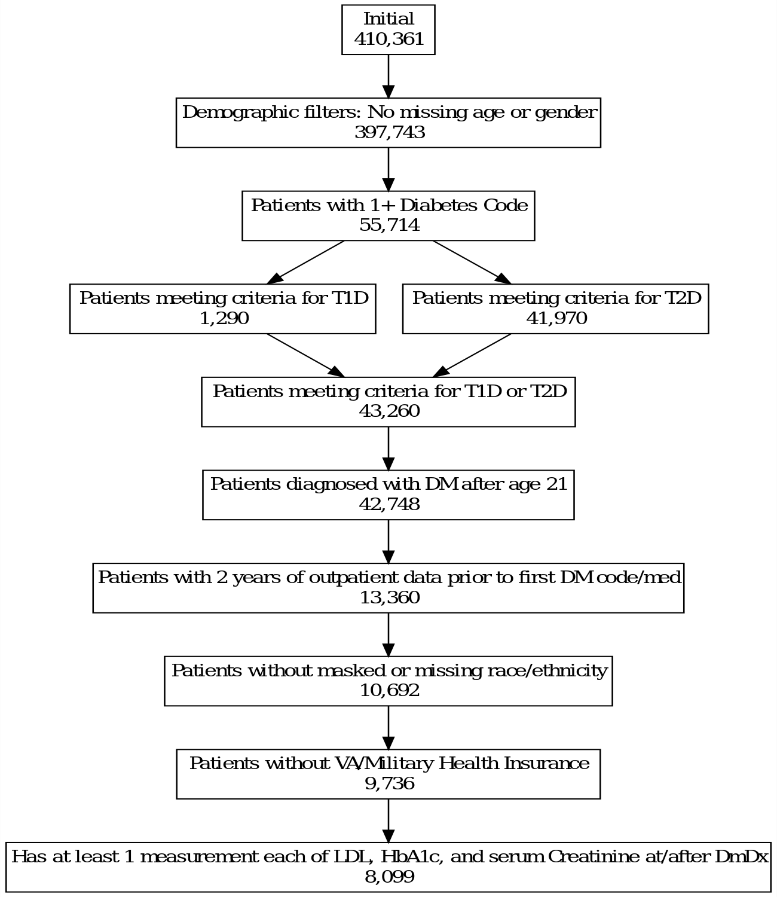


The final count refers to the number of patients at the index date (i.e., the date of the first diabetes diagnosis). Final patient counts used for model fitting vary by landmark and require patients to be alive and without end-stage renal disease at the time of the landmark.

DM: Diabetes Mellitus; DM Dx: Diabetes mellitus diagnosis; HbA1c: Hemoglobin A1C; LDL: Low-density lipoprotein; Med: Medication; VHA: Veterans Health Administration; T1D: Type 1 Diabetes; T2D: Type 2 Diabetes;
