# Supplementary material for: Dynamically predicting renal failure after development of diabetes across biobanks
Source: PLOS Digit Health. 2026 May 4;5(5):e0001375. doi: 10.1371/journal.pdig.0001375 (PMC13138643; doi:10.1371/journal.pdig.0001375)
Supplement: S2 Fig — (DOCX) [file pdig.0001375.s004.docx]

# **S2 Fig.**

Cumulative incidence function (CIF) for **A**. End-Stage Renal Disease (ESRD); **B**. Death in VHA and its selected subpopulations across different landmark times.

**A.** ESRD **B.** Death


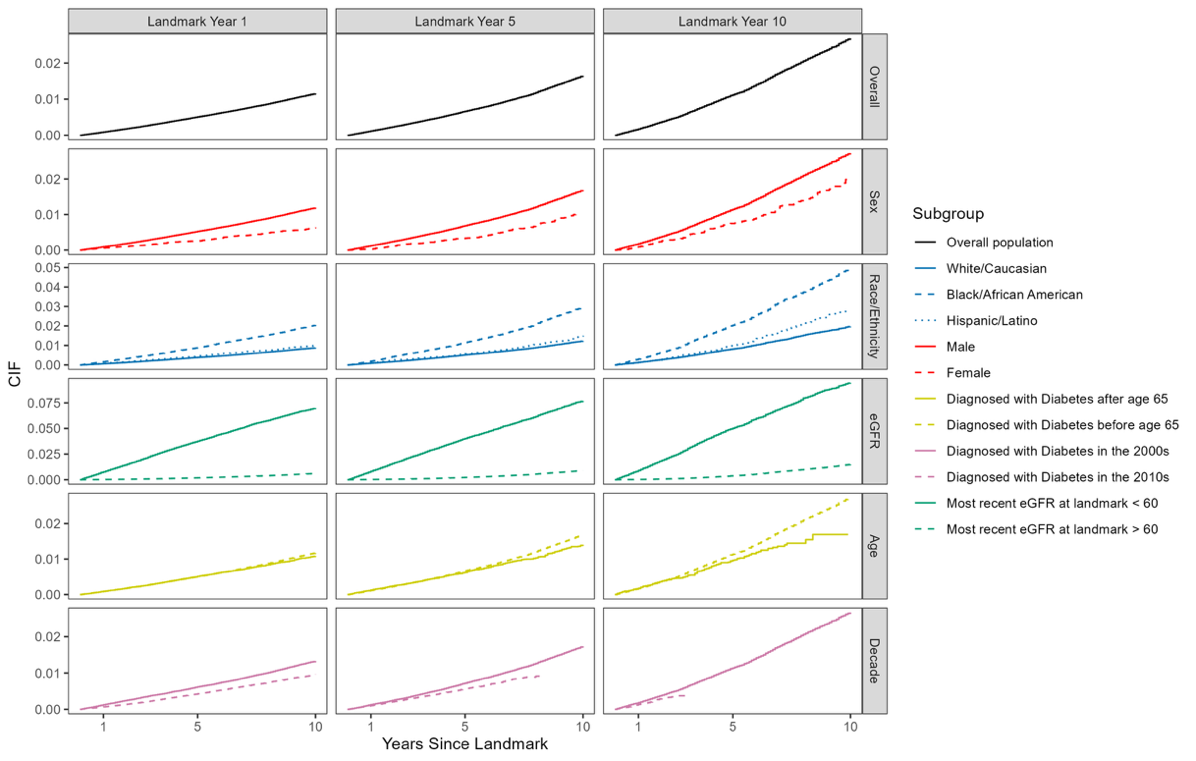

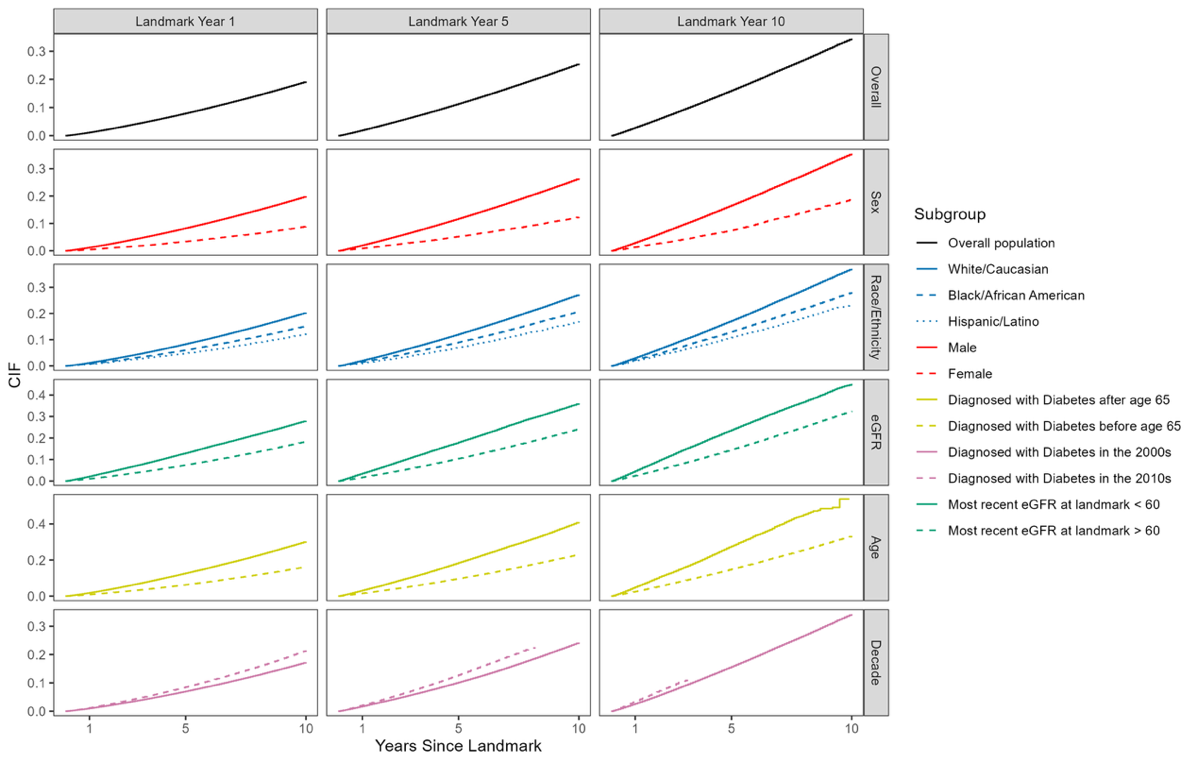


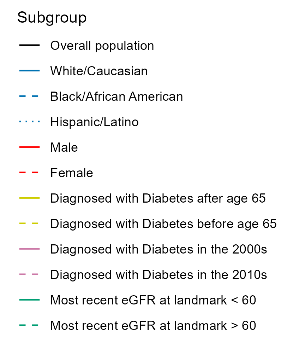


eGFR: Estimated glomerular filtration rate; VHA: Veterans Health Administration.
