# Supplementary material for: Dynamically predicting renal failure after development of diabetes across biobanks
Source: PLOS Digit Health. 2026 May 4;5(5):e0001375. doi: 10.1371/journal.pdig.0001375 (PMC13138643; doi:10.1371/journal.pdig.0001375)
Supplement: S3 Fig — (DOCX) [file pdig.0001375.s005.docx]

# **S3 Fig.**

Distribution (median, 25th, and 75th percentile) of biomarker measures extracted from VHA, AoU, separated by sex.

*
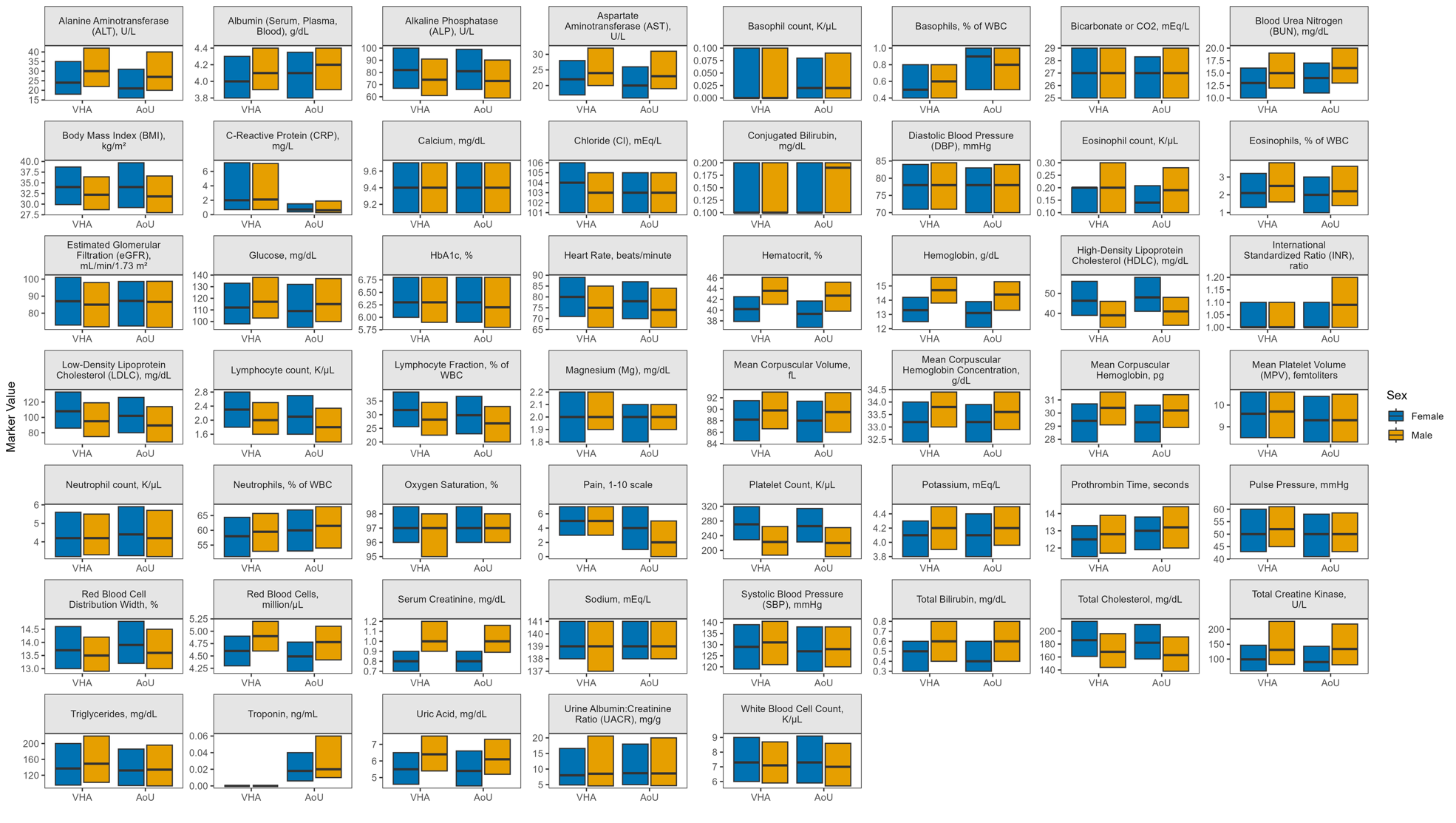
*

AoU: All of Us; VHA: Veterans Health Administration
