# Supplementary material for: Dynamically predicting renal failure after development of diabetes across biobanks
Source: PLOS Digit Health. 2026 May 4;5(5):e0001375. doi: 10.1371/journal.pdig.0001375 (PMC13138643; doi:10.1371/journal.pdig.0001375)
Supplement: S4 Fig — (DOCX) [file pdig.0001375.s006.docx]

# **S4 Fig.**

Distribution (median, 25th, and 75th percentile) of the number of repeated measurements of biomarkers extracted from VHA, AoU, separated by sex.


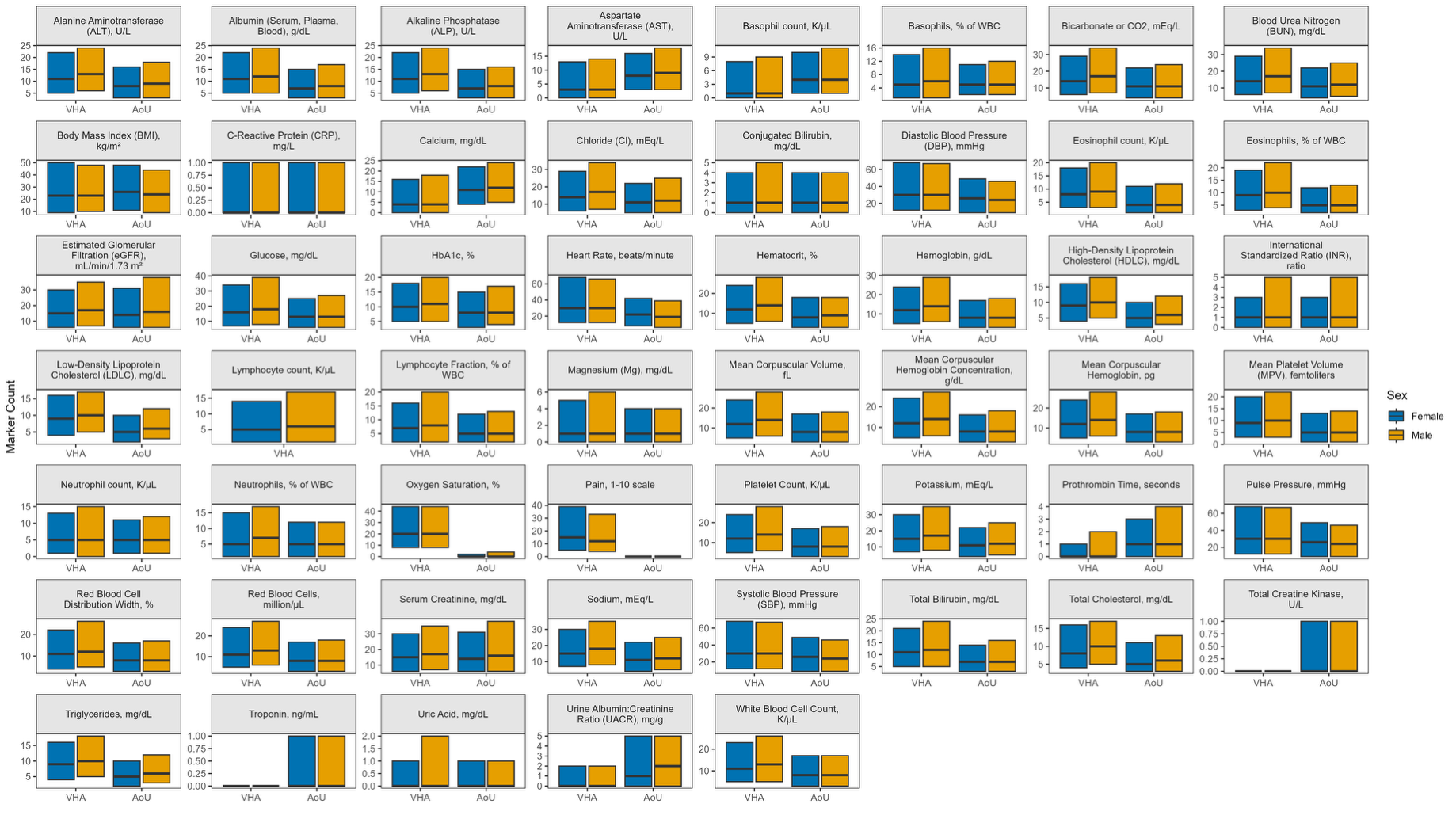


For the 3-year period, i.e., 2 years before DM Dx and 1 year after DM Dx (i.e., LM1), a maximum of 1 measurement is counted. For the period after LM1, all measurements are counted. For the period prior to 2 years before DM Dx, no measurements are counted.

AoU: All of Us; DM Dx: Diabetes diagnosis; LM: landmark; VHA: Veterans Health Administration
