# Supplementary material for: Dynamically predicting renal failure after development of diabetes across biobanks
Source: PLOS Digit Health. 2026 May 4;5(5):e0001375. doi: 10.1371/journal.pdig.0001375 (PMC13138643; doi:10.1371/journal.pdig.0001375)
Supplement: S5 Fig — (DOCX) [file pdig.0001375.s007.docx]

# **S5 Fig.**

Calibration plots at three landmark times and for three horizons in VHA.


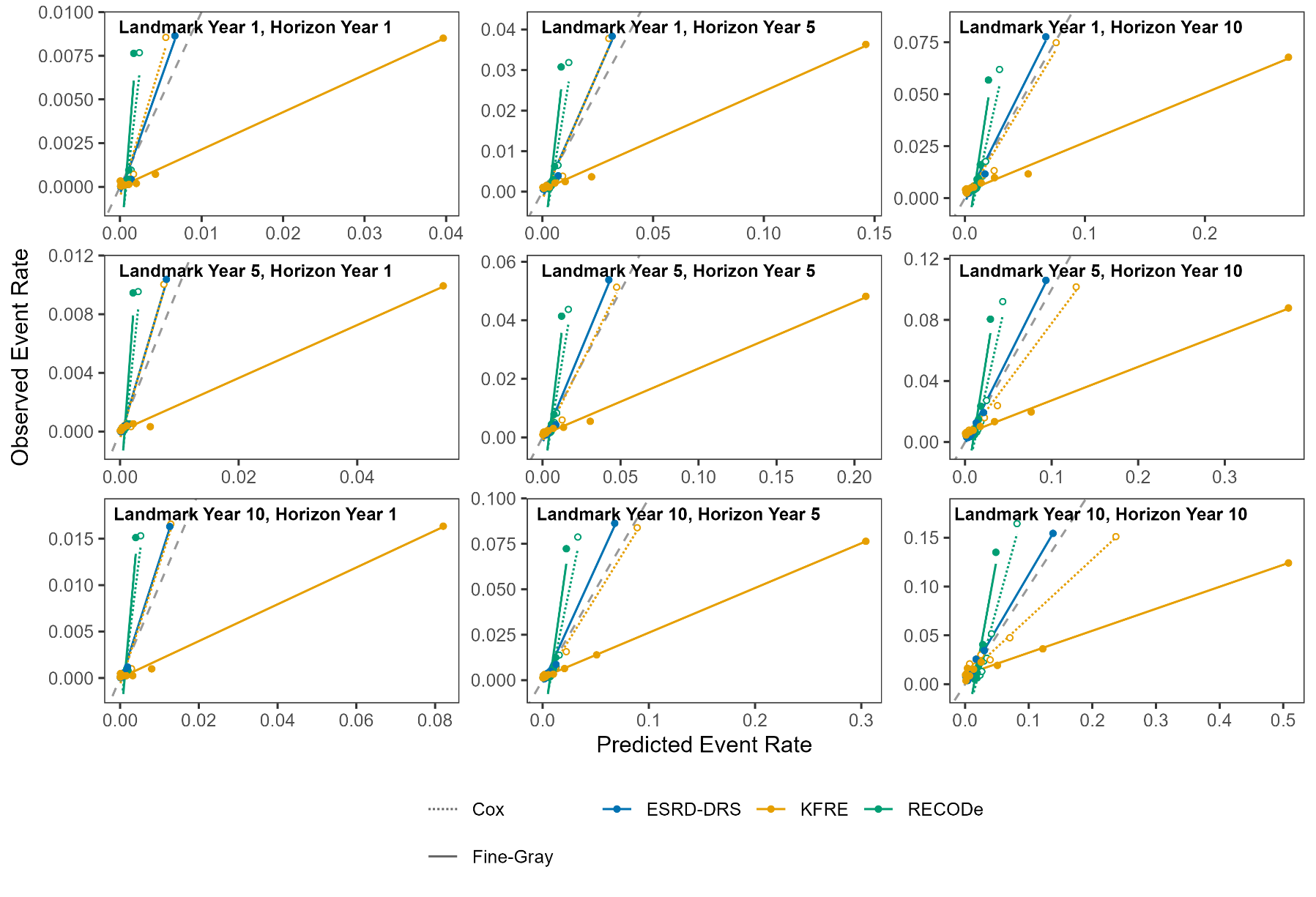


X-axes refer to deciles of predicted risk based on the ESRD-DRS and their corresponding observed pseudo-event rates in VHA.

ESRD-DRS: End-Stage Renal Disease dynamic risk score; VHA: Veterans Health Administration; KFRE: Kidney Failure Risk Equation; RECODe: Risk Equations for Complications Of type 2 Diabetes.
