# Supplementary material for: Dynamically predicting renal failure after development of diabetes across biobanks
Source: PLOS Digit Health. 2026 May 4;5(5):e0001375. doi: 10.1371/journal.pdig.0001375 (PMC13138643; doi:10.1371/journal.pdig.0001375)
Supplement: S6 Fig — (DOCX) [file pdig.0001375.s008.docx]

# **S6 Fig.**

Calibration plots at three landmark times and for three horizons in AoU.


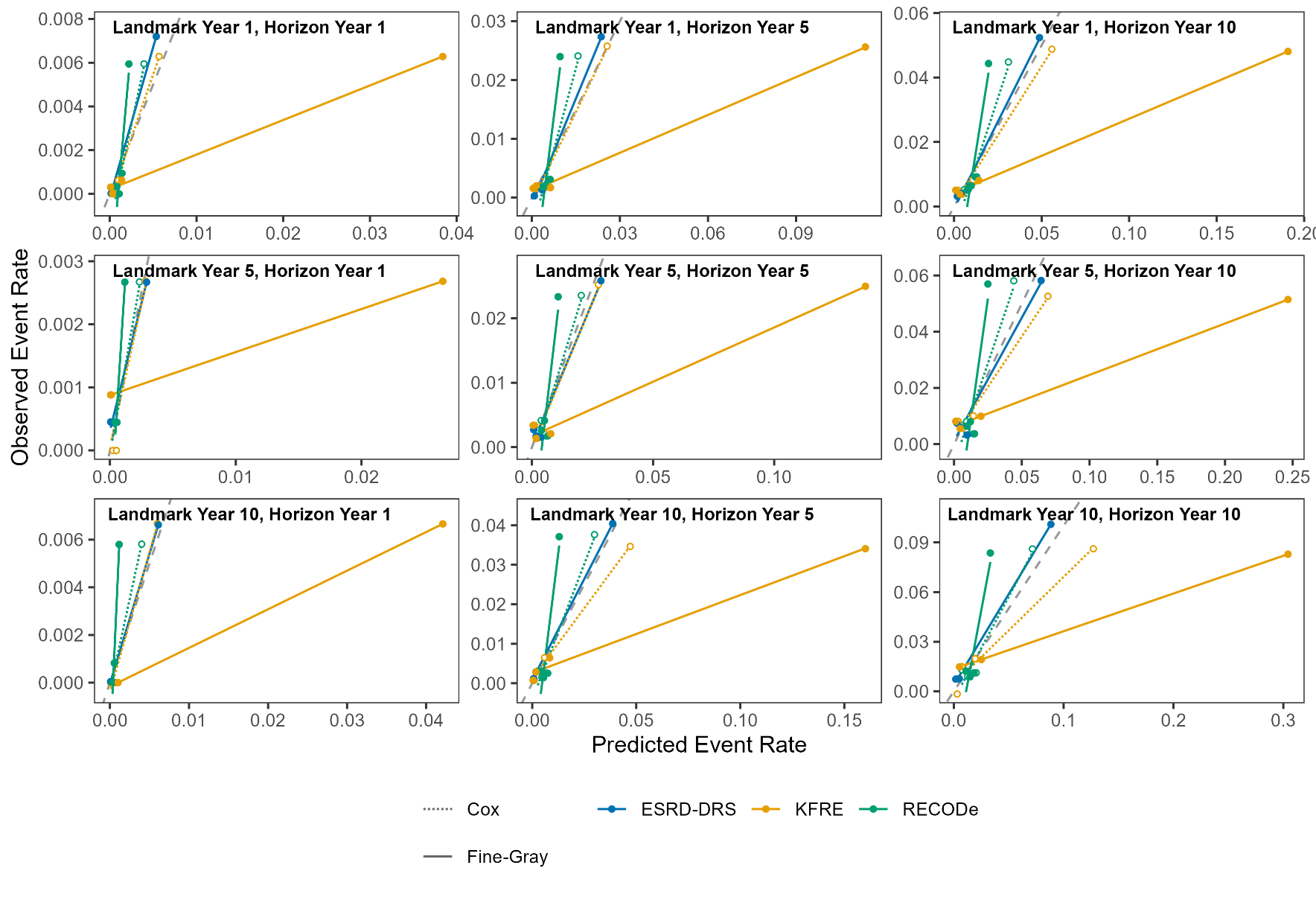


Quartile of predicted probability of ESRD is plotted against corresponding pseudo-event rates in AoU.

ESRD: End-Stage Renal Disease; AoU: All of Us; ESRD-DRS: End-Stage Renal Disease dynamic risk score; KFRE: Kidney Failure Risk Equation; RECODe: Risk Equations for Complications Of type 2 Diabetes.
