# Supplementary material for: Dynamically predicting renal failure after development of diabetes across biobanks
Source: PLOS Digit Health. 2026 May 4;5(5):e0001375. doi: 10.1371/journal.pdig.0001375 (PMC13138643; doi:10.1371/journal.pdig.0001375)
Supplement: S7 Fig — (DOCX) [file pdig.0001375.s009.docx]

# **S7 Fig.**

Calibration plots at three landmark times and for three horizons among select VHA subpopulations

**A**: Subgroups except eGFR <60. **B**: subgroup with eGFR < 60.


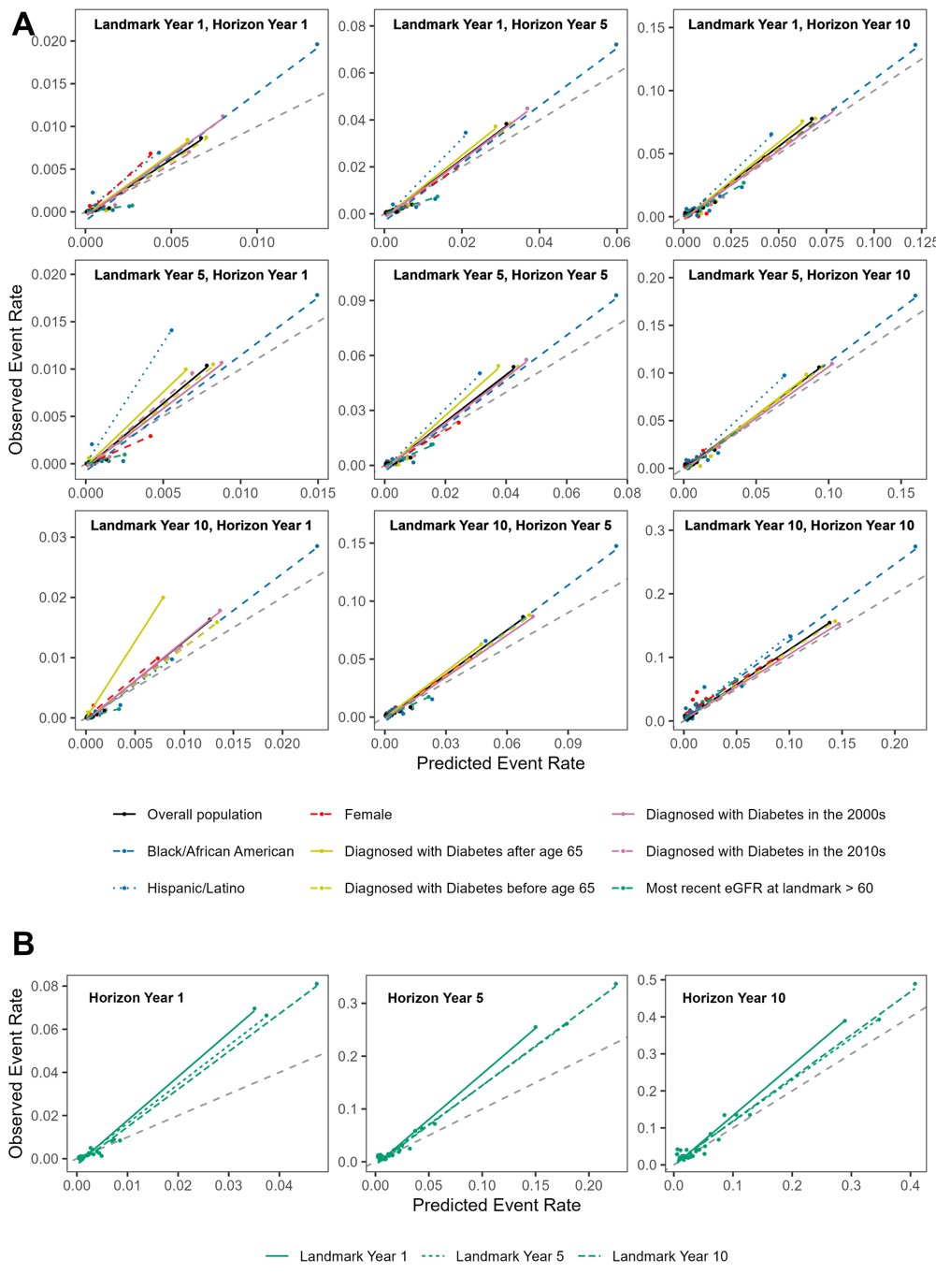


Decile of predicted probability of ESRD is plotted against corresponding pseudo-event rates.

eGFR: Estimated glomerular filtration rate; ESRD: End-Stage Renal Disease; VHA: Veterans Health Administration.
