# Supplementary material for: Dynamically predicting renal failure after development of diabetes across biobanks
Source: PLOS Digit Health. 2026 May 4;5(5):e0001375. doi: 10.1371/journal.pdig.0001375 (PMC13138643; doi:10.1371/journal.pdig.0001375)
Supplement: S8 Fig — (DOCX) [file pdig.0001375.s010.docx]

# **S8 Fig.**

AUC (i.e., AUROC) (95% CI, shaded) of dynamic prediction (blue), KFRE (yellow), and RECODe model (green) for ESRD in VHA among select subpopulations.


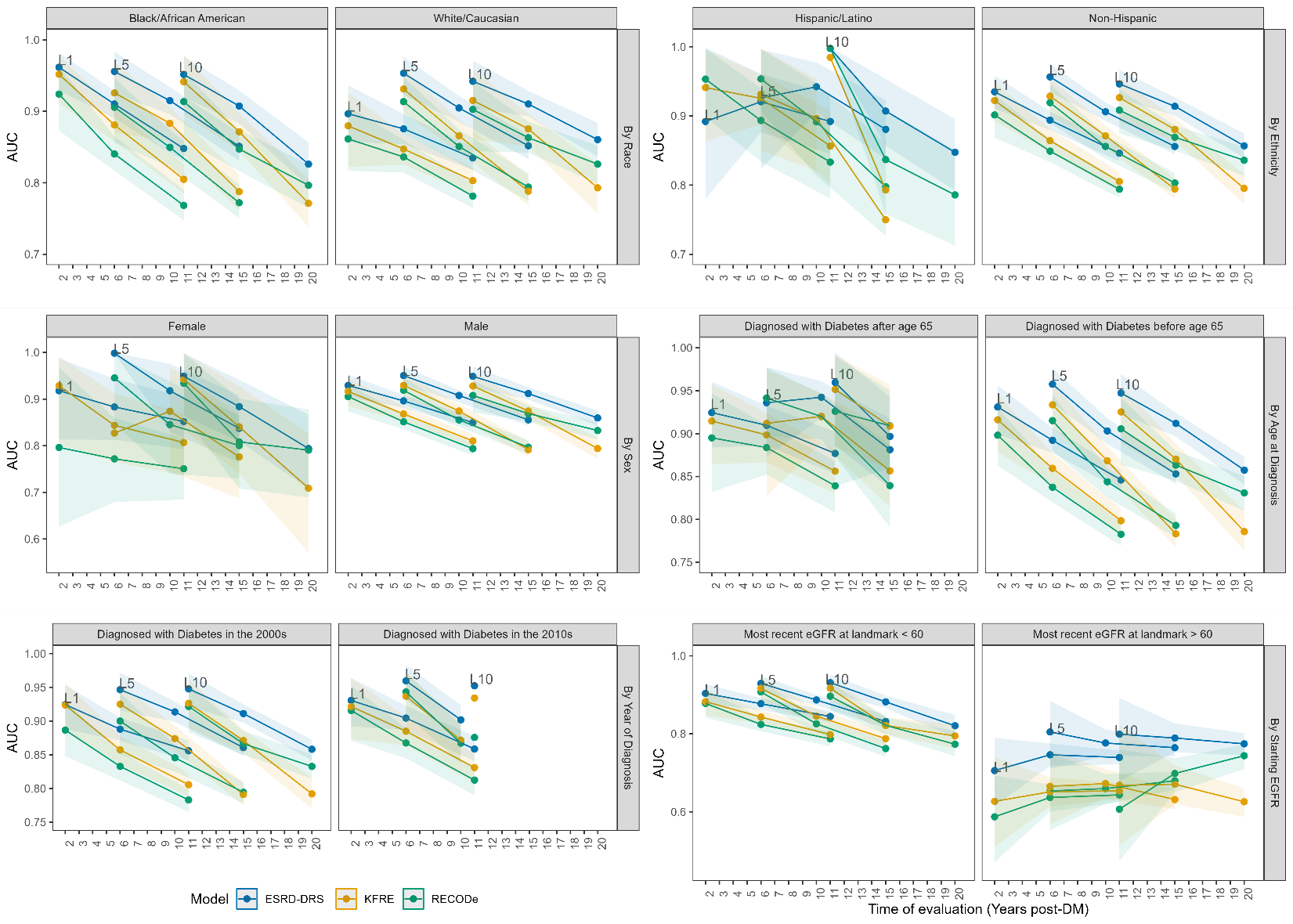


AUROC: Area Under the Receiver Operating Characteristic curve; ESRD: End Stage Renal Disease; KFRE: Kidney Failure Risk Equation; RECODe: Risk Equations for Complications Of type 2 Diabetes; VHA: Veterans Health Administration
