# Supplementary material for: Dynamically predicting renal failure after development of diabetes across biobanks
Source: PLOS Digit Health. 2026 May 4;5(5):e0001375. doi: 10.1371/journal.pdig.0001375 (PMC13138643; doi:10.1371/journal.pdig.0001375)
Supplement: S10 Fig — (DOCX) [file pdig.0001375.s012.docx]

# **S10 Fig.**

SHAP values for top 10 features in VHA across three landmark models and three horizon times.


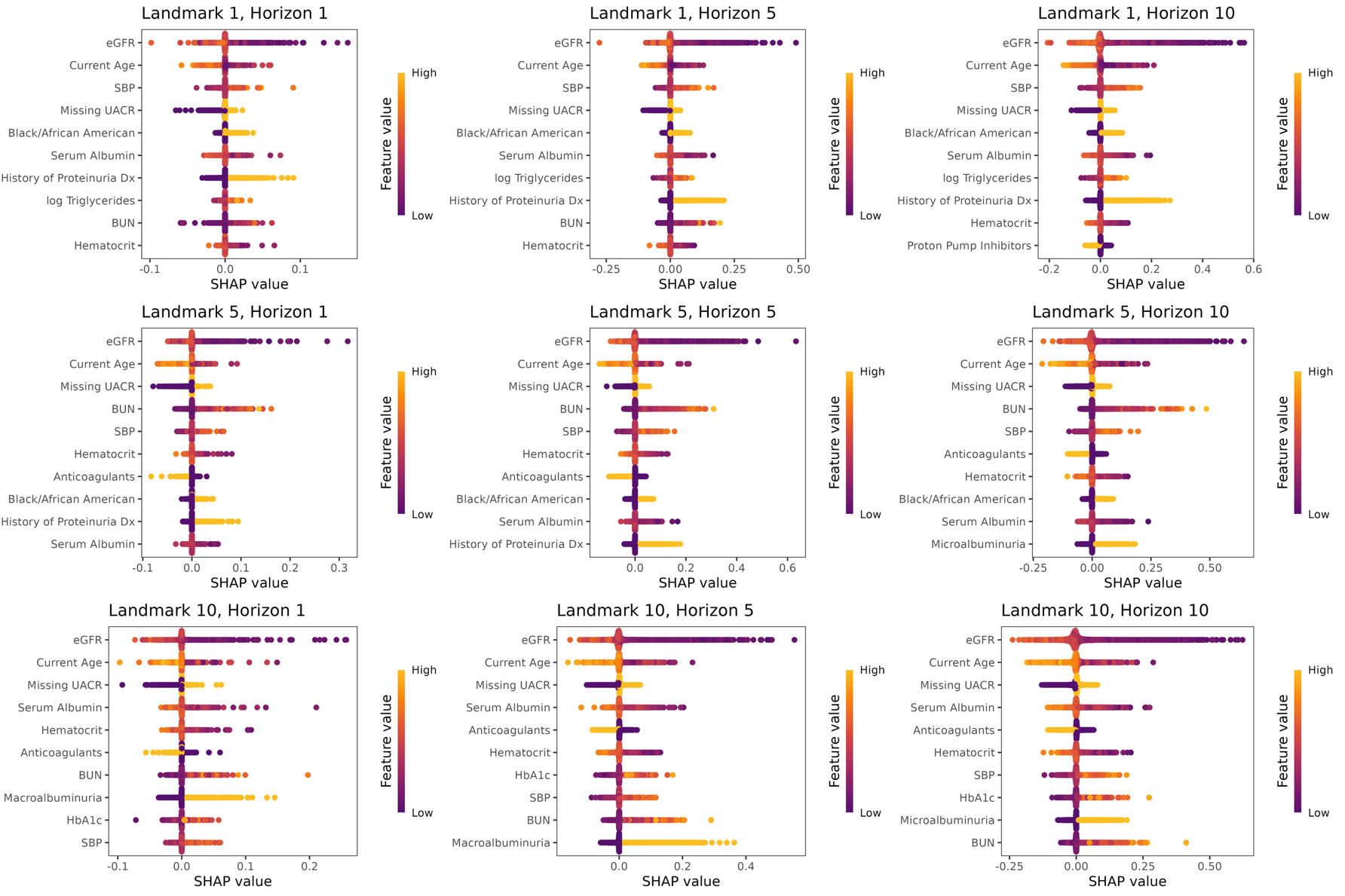


Blood Urea Nitrogen: BUN; Dx: Diagnosis; UACR: Urine Albumin-to-Creatinine Ratio; eGFR: Estimated glomerular filtration rate; HbA1c: Hemoglobin A1C; SBP: Systolic blood pressure; SHAP: SHapley Additive exPlanations.
